# Minding the margins: Evaluating the impact of COVID-19 among Latinx and Black communities with optimal qualitative serological assessment tools

**DOI:** 10.1371/journal.pone.0307568

**Published:** 2024-07-25

**Authors:** Raquel A. Binder, Angela M. Matta, Catherine S. Forconi, Cliff I. Oduor, Prajakta Bedekar, Paul N. Patrone, Anthony J. Kearsley, Boaz Odwar, Jennifer Batista, Sarah N. Forrester, Heidi K. Leftwich, Lisa A. Cavacini, Ann M. Moormann

**Affiliations:** 1 Department of Medicine, University of Massachusetts Chan Medical School, Worcester, MA, United States of America; 2 Department of Pathology and Laboratory Medicine, Warren Alpert Medical School, Brown University, Providence, RI, United States of America; 3 Applied and Computational Mathematics Division, National Institute of Standards and Technology, Gaithersburg, MD, United States of America; 4 Department of Applied Mathematics and Statistics, Johns Hopkins University, Baltimore, MD, United States of America; 5 Department of Population and Quantitative Health Sciences, University of Massachusetts Chan Medical School, Worcester, MA, United States of America; 6 Department of Obstetrics and Gynecology, University of Massachusetts Chan Medical School, Worcester, MA, United States of America; US Environmental Protection Agency, UNITED STATES OF AMERICA

## Abstract

COVID-19 disproportionately affected minorities, while research barriers to engage underserved communities persist. Serological studies reveal infection and vaccination histories within these communities, however lack of consensus on downstream evaluation methods impede meta-analyses and dampen the broader public health impact. To reveal the impact of COVID-19 and vaccine uptake among diverse communities and to develop rigorous serological downstream evaluation methods, we engaged racial and ethnic minorities in Massachusetts in a cross-sectional study (April—July 2022), screened blood and saliva for SARS-CoV-2 and human endemic coronavirus (hCoV) antibodies by bead-based multiplex assay and point-of-care (POC) test and developed across-plate normalization and classification boundary methods for optimal qualitative serological assessments. Among 290 participants, 91.4% reported receiving at least one dose of a COVID-19 vaccine, while 41.7% reported past SARS-CoV-2 infections, which was confirmed by POC- and multiplex-based saliva and blood IgG seroprevalences. We found significant differences in antigen-specific IgA and IgG antibody outcomes and indication of cross-reactivity with hCoV OC43. Finally, 26.5% of participants reported lingering COVID-19 symptoms, mostly middle-aged Latinas. Hence, prolonged COVID-19 symptoms were common among our underserved population and require public health attention, despite high COVID-19 vaccine uptake. Saliva served as a less-invasive sample-type for IgG-based serosurveys and hCoV cross-reactivity needed to be evaluated for reliable SARS-CoV-2 serosurvey results. The use of the developed rigorous downstream qualitative serological assessment methods will help standardize serosurvey outcomes and meta-analyses for future serosurveys beyond SARS-CoV-2.

## Introduction

Differential health care access and exposure risks have led to racial and ethnic COVID-19 disparities in the United States (US), leaving Latinx and Black communities to experience disproportionately high SARS-CoV-2 infection rates and COVID-19 related morbidity and mortality [1, 2]. Seroprevalence studies have become essential public health tools to assess the regional spread and pre-existing immunity to SARS-CoV-2 among at-risk populations [3–5]. Further, by distinguishing between anti-SARS-CoV-2 spike (S), receptor-binding domain (RBD), and nucleocapsid (N) antibodies, COVID-19 vaccine uptake (RBD/S only) can be estimated and compared to past SARS-CoV-2 infections [6]. Anti-SARS-CoV-2 immunoglobulin (Ig)G and IgA antibodies can be measured in both blood and saliva, the latter serving as less invasive sample collection alternative [7].

Here, we engaged ethnic and racial minorities to evaluate the impact of COVID-19 in the Greater Worcester area from April to July of 2022 and assessed blood- and saliva-based serosurvey methods. Overall, this study (i) evaluated the impact of COVID-19 and vaccine uptake among marginalized communities, (ii) confirmed the utility of using saliva for serosurveys, (iii) compared the utility of a bead-based multiplex assay vs. a point-of-care (POC) test for SARS-CoV-2 antibody measurements, and (iv) demonstrated the benefit of developing and using classification boundary methods for optimal interpretation of serological assays.

## Methods

### Participant recruitment and sample collection

This cross-sectional study was approved by the University of Massachusetts Chan Medical School (UMass Chan) Institutional Review Board (IRB Docket # H00023083). Structured interviews with Black and Latinx community members in the Greater Worcester Area in Massachusetts (MA) were conducted to identify recruitment barriers for participation in research studies. IRB-approved study flyers were distributed prior to engagement with local stakeholders. We joined regular community gatherings organized by Net of Compassion, Centro Hispano, and Central MA YMCA in Worcester, along with the St. John Catholic Church in Clinton for in-person recruitment events implemented both in Spanish and English from April 21^st^ to July 4^th^ of 2022. On site, we provided a fact sheet explaining the purpose of the study and were available to answer questions in Spanish and English. We obtained informed verbal consent from eligible individuals (inclusion criteria: 18+ years of age, exclusion criteria: prisoners and people unable to communicate in English or Spanish) and participants were asked to fill out a brief RedCap survey covering demographic information, COVID-19 vaccine, and SARS-CoV-2 infection history through tablets that were provided by the study team or by QR codes that could be scanned on personal devices. We used verbal (not written) consent for this minimal risk study due to hesitation of documentation and decreased literacy rate among individuals from underserved communities. The verbal consent was documented by the research team member interviewing the participant and this method was approved by the IRB prior to study begin. Blood and saliva samples were collected with Tasso SST devices (Tasso, Inc., Seattle, WA) and SuperSal2^®^ devices (Oasis Diagnostics, Vancouver, WA), respectively, as per manufacturer’s guidelines. SARS-CoV-2 anti-immunoglobulin (Ig)G and IgM antibodies were measured with an emergency use authorized approved POC test (FaStep from Assure Tech, Hangzhou, China), which detects both anti-SARS-CoV-2 N and S antibodies. The POC test results were provided to participants immediately, along with a $50 reimbursement. See Supplemental Methods for more information on sample collection and processing.

### Multiplex Luminex assay

The following SARS-CoV-2 antigens were coupled to Luminex MagPlex Microspheres as indicated by the manufacturer: Wild-type (WT; Wuhan) full-length spike (S), WT nucleocapsid (N), WT receptor-binding domain (RBD), RBD alpha, RBD beta, RBD gamma, RBD delta, RBD lambda, and RBD omicron. Following human endemic coronavirus (hCoV) antigens and a Bovine Serum Albumin (BSA) control were coupled: HKU1, OC43, NL63, and 229E, see Supplemental Methods for more details. Briefly, after validation of conjugated beads, the participant samples were screened on 96- or 384-well plates, including a seven- or ten-point serial dilution standard. Conjugated beads covering the antigen panel were combined and washed, incubated, washed again, and biotinylated anti-human secondary IgG or IgA (BD Pharmingen) antibody added. After incubation and washing, phycoerythrin conjugated streptavidin was added (BD Pharmingen). Finally, after incubation, washing, and resuspension, the plate was screened by a FlexMap 3D Luminex instrument. The antigen-specific median fluorescence intensity (MFI) for each sample was recorded and BSA subtracted (including for the standards) to account for non-specific bead binding [8]. Saliva samples were screened for total IgG and total IgA antibodies to account for differential salivation flow rates by coupling anti-human IgG gamma chain (Bio-Rad, Hercules, CA) and anti-human IgA alpha chain protein (Abcam, Cambridge, UK) respectively.

Previously described de-identified banked blood samples served as positive (n = 50) and negative blood controls (n = 50) [9]. Banked saliva samples (n = 50), collected with the same SuperSal2 devices, served as alternative control group for the seroprevalence calculation, see “Qualitative Serological Assessments” section below.

### Across-plate normalization

Dilution series of standards for each antigen-plate combination were weighted using a plate-dependent variance while a normalization factor was computed with custom MATLAB scripts. This factor was then applied by multiplying it with each antigen/isotype-specific sample MFI on the plate, see Supplemental Methods for more details.

### Qualitative serological assessments

Sample MFIs were translated to qualitative (i.e., binary positive/negative) outcomes as described in reference [10] and in the Supplemental Methods. Briefly, for the blood samples, empirical training data were taken as approximate probability models of measurement outcomes for each antigen, conditioned on knowing the class of the underlying sample. The analysis was applied to multidimensional data by treating up to three antigens as distinct axes in a coordinate space, see **S1 Fig** for a three-dimensional analysis example. Due to the lack of collection method- and population-matched controls, the saliva-based IgG seroprevalence calculations were determined with alternative control samples from a 2020 Kenyan study (proxy for negative training data), see Supplemental Methods for more details.

### Statistical analysis

Statistical calculations and graphs were done in Prism v9.4.1, R v2023.09.1+494, and MATLAB R2023a Update 5 (9.14.0.2337262).

## Results

### Demographics and vaccine/infection history

A total of 290 adult study participants were enrolled in Worcester, Shrewsbury, and Clinton, MA between April and July of 2022. Most participants donated blood (98.6%, n = 286) and saliva (94.8%, n = 275) samples. Participants who did not give a saliva sample mostly lacked saliva production (i.e., dry mouth), especially among elderly, but were not hesitant to donate the sample. The demographic, clinical, and SARS-CoV-2 infection history survey was filled out by 98.3% participants (n = 285), while 47.6% (n = 138) chose to answer in Spanish and 51.7% (n = 150) in English (see Supplemental Materials for full survey). Most participants self-identified as Latinx/Hispanic (67.6%, n = 196), and female (61.0%, n = 177), while the average age was 45 years (range: 18–82, STDV: 16.3), and 31.4% (n = 91) of participants received a college or higher education degree, see **Table 1**. Non-Hispanic White participants constituted 13.4% (n = 39) of the study population. Among the participants, 15.5% (n = 45) reported pre-existing health conditions, most commonly hypertension, obesity, diabetes type II, and asthma, while 13.5% (n = 39) reported smoking or vaping prior to the pandemic, see **S1 Table** in S1 File. Of note, the self-reported pre-existing health conditions (particularly the high prevalence of hypertension, diabetes, and asthma) reflect health-related risk factors reported among US minorities in other studies and increase the vulnerability to COVID-19 associated complications [11–13].

**Table 1:** Study participant demographics from 290 study participants enrolled in Massachusetts from April to July 2022 and 286 associated blood samples collected during the study period.

	Overall	Nucleocapsid IgG *Positive*	Nucleocapsid IgG *Negative*		χ^2^
		Serum	Serum		
	n (%)	n (%)	n (%)		p-value^∋^
Total	290 (100)	136 (49.9±7)^#^		150 (52.5)	
**Age** (years)					
18–40	126 (43.5)	52 (38.2)	72 (48.0)		0.292
41–60	96 (33.1)	45 (33.1)	51 (34.0)		
60	59 (20.3)	31 (22.8)	26 (17.3)		
Missing	9 (3.1)	8 (5.9)	1 (0.7)		
**Gender**					
Female	177 (61.0)	79 (58.1)	96 (64.0)		0.393
Male	109 (37.6)	54 (39.7)	53 (35.3)		
Non-binary	3 (1.0)	2 (1.5)	1 (0.7)		
Missing	1 (0.4)	1 (0.7)	0 (0.0)		
**Race**					
White	97 (33.5)	39 (28.7)	56 (37.3)		0.039*
Mixed/Mestizo	90 (31.0)	53 (39.0)	37 (24.7)		
Black/African American	29 (10.0)	14 (10.3)	15 (10.0)		
Asian	31 (10.7)	9 (6.6)	21 (14.0)		
Other	43 (14.8)	21 (15.4)	21 (14.0)		
**Ethnicity**					
Hispanic	196 (67.6)	104 (76.5)	90 (60.0)		0.0013**
non-Hispanic	91 (31.4)	29 (21.3)	60 (40.0)		
Missing	3 (1.0)	3 (2.2)	0 (0.0)		
**Education**					
High School or less	100 (34.5)	57 (41.9)	40 (26.7)		0.0193*
College or more	91 (31.4)	37 (27.2)	54 (36.0)		
Missing	99 (34.1)	42 (30.9)	56 (37.3)		
**SARS-CoV-2 + Test**					
Yes	121 (41.7)	71 (52.2)	49 (32.7)		0.0003***
No	163 (56.2)	59 (43.4)	101 (67.3)		
Missing	6 (2.1)	6 (4.4)	0 (0.0)		
**Fully Recovered** ^ϕ^					
Yes	108 (37.2)	54 (39.7)	53 (35.3)		0.0099**
No	21 (7.2)	18 (13.3)	3 (2.0)		
Long COVID	19 (6.6)	12 (8.8)	7 (4.7)		
Never had COVID	136 (46.9)	46 (33.8)	87 (58.0)		
Missing	6 (2.1)	6 (4.4)	0 (0.0)		

^∋^The “Missing” categories, the “Non-binary” category in gender, and the “Never had COVID” category under “Fully Recovered” were omitted for the χ^2^ test comparison; p-value < 0.05. The gender, ethnicity, education, and SARS-CoV-2 test comparison were done with the Fisher’s exact test (2x2 categories). * p-value < 0.05, ** p-value < 0.01, ***p-value < 0.001.

^#^136 is the empirical count of positives (equal to a 47.5% empirical seroprevalence) and 49.9% (95% CI: ±7) is the calculated bias-corrected seroprevalence.

^ϕ^COVID-19 recovery among all study participants (including 121 test-confirmed cases). Self-reporting having fully recovered from a SARS-CoV-2 infections (“Yes”) or not (“No”), whether infection-associated symptoms lasted past 4 weeks of the initial infection and therefore reported long COVID-19 symptoms (Long COVID), and those who never had COVID-19 (Never had COVID). For the χ^2^ test comparison the “Missing” and the “Never had COVID” categories were omitted.

A total of 265 (91.4%) participants received at least one dose of a COVID-19 vaccine; 45.7% (n = 121) Moderna; 40.0% (n = 106) Pfizer; 8.6% (n = 23) Johnson and Johnson; and 5.7% (n = 15) ‘other’, for the base vaccine series, see **S1 Table** in S1 File. The vaccine uptake was lower for the second dose (84.5%, n = 245) and booster (68.3%, n = 198) while most participants received an influenza vaccine in the past 5 years (79.3%, n = 230). As for vaccine-related side effects, 37.7% (n = 100), 43.7% (n = 107), and 35.9% (n = 71) of participants did not experience any post-COVID-19 vaccine symptoms post-first dose, -second dose and -booster, respectively. Among those who experienced vaccine-associated symptoms post-base vaccine series and booster, the average severity scores were 4.1 (STDV: 2.4; range: 1–10) and 3.6 (STDV: 2.2; range:1–10) respectively on a scale from 1 to 10, while arm soreness, fever, and fatigue were the most frequently reported symptoms. Further, 16.6% (n = 44), 14.7% (n = 36), and 6.1% (n = 12) of participants experienced severe symptoms (rating ≥ 6) post-first dose, second dose, and booster, respectively, encompassing thrombosis, strokes, fainting, and migraines. A total of 6 participants reported vaccine-associated hospitalizations, encompassing all doses for all participants. Among those who were not vaccinated, participants reported being hesitant because of fear of COVID-19 vaccine side effects, lack of trust in the vaccine, and not knowing enough about the vaccine.

A total of 121 (41.7%) participants reported testing positive for SARS-CoV-2 at least once and the associated average symptom severity score was 5.4 (STDV: 2.4; range: 1–10), while 43.0% (n = 52) experienced severe symptoms (rating ≥ 6) and 9.1% (n = 11) were hospitalized. The most common symptoms were body aches (52.1%, n = 63), fever (51.2%, n = 62), fatigue (51.2%, n = 62), cough (48.8%, n = 59), headache (46.3%, n = 56), sore throat (45.5%, n = 55), congestion/runny nose (38.0%, n = 46), and loss of smell or taste (36.4%, n = 44). A total of 7 individuals (5.8%) reported not having any symptoms associated with the positive test (i.e., asymptomatic infections). There was an association between reporting pre-existing health conditions and elevated severity scores (rating ≥ 6, Fisher’s exact test, p = 0.0475) but not between smoking/vaping and elevated severity scores (rating ≥ 6, Fisher’s exact test, p = >0.999). Among those with confirmed SARS-CoV-2 infections (n = 121), 26.5% (n = 32) reported not having fully recovered from the infection and 14.1% (n = 17) reported long COVID-19 symptoms (persistent symptoms past 4 weeks of diagnosis). For further analysis, the categories “not fully recovered from the infection” and reporting “long COVID-19 symptoms” were collapsed. Within this category, 78.1% (n = 25) were female, 87.5% (n = 28) were Latinx, 59.4% (n = 19) of mixed/mestizo race, and the average age was 48.6 years (STDV: 16.4, range: 19–82). There was a significant association between being 50+ years of age and not having fully recovered from COVID-19 symptoms (Fisher’s exact test, p = 0.0135). There was no significant correlation between being female or reporting pre-existing health conditions and not having recovered from COVID-19 symptoms. Since the majority of the study population self-identified as Latinx and/or belonging to racial minorities, we were not able to contrast unresolved COVID-19 symptoms or any other variables among racial/ethnic sub-groups.

### Blood-based SARS-CoV-2 antibodies

**Fig 1:**
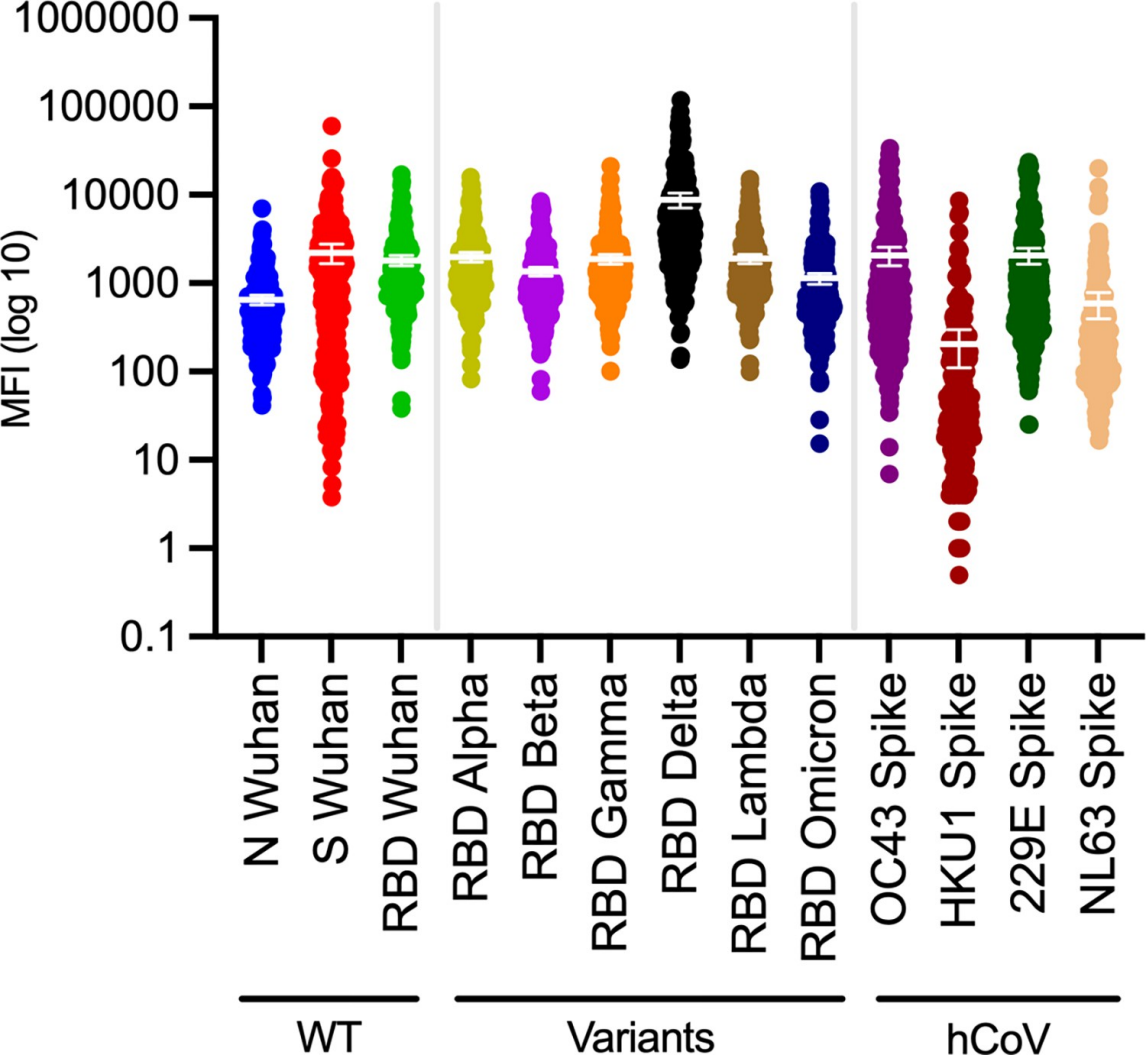
Serum IgG outcome distribution. Dot plot with means and 95% confidence intervals (CI) of antigen-specific outcomes (MFI—BSA) for serum IgG, covering SARS-CoV-2 (including variants) and human endemic coronaviruses (OC43, HKU1, NL63, 229E) with log10 y-axis. See **S2 Fig** for plots with linear y-axis. MFI, median fluorescence intensity. N, Nucleocapsid. S, Spike. RBD, Receptor Binding Protein. hCoV, human endemic coronaviruses. WT, wild-type (Wuhan).

Among those who received a SARS-CoV-2 POC antibody results (n = 284), 93.7% (n = 266) were positive for IgG and 4.6% (n = 13) for IgM. The POC test covered both SARS-CoV-2 N and S antibodies together, while the multiplex assay allowed measuring presence of antibodies based on individual antigens and therefore distinguish between vaccine- (S/RBD-only since all currently FDA-approved COVID-19 vaccines in the US are S/RBD-based and do not include the N antigen) and infection-induced antibodies. The serum multiplex-based *IgG* seroprevalences resulted in 97.5% ± 2.4% (95% CI) for RBD/S/N (3 antigens), 99.9% ± 3.4% (95% CI) for RBD/S (2 antigens), 97.2% ± 2.0% (95% CI) for S/N (2 antigens), 96.5% ± 2.2% (95% CI) for RBD/N (2 antigens), 96.5% ± 2.2% (95% CI) for RBD, and 97.9% ± 1.7% (95% CI) for S (see **S2 Table** in S1 File), mirroring the high self-reported vaccination uptake. The serum-based IgG N seroprevalence was 49.9% ± 7.0% (95% CI), indicating past infection rather than vaccination, similar to the percent of self-reported exposures. Statistically significant differences in N-specific serological test outcomes were observed among study participants based on race, ethnicity, education, and having fully recovered from COVID-19 symptom, as well as based on self-reported SARS-CoV-2 test results prior to study participation, see **Table 1**. The S- and RBD-specific serological test outcomes were not contrasted based on the listed categories due to the high overall seroprevalence and self-reported vaccine uptake being close to 100%. As for SARS-CoV-2 variants, the delta variant had the highest mean MFI and reached the highest maximum MFI read, see **Fig 1**. While antigen-specific MFI are influenced by antigen quality and steric hindrance this may reflect that SARS-CoV-2 delta variant antibodies were the most abundant among our study population at the time of sample collection.

**Fig 2:**
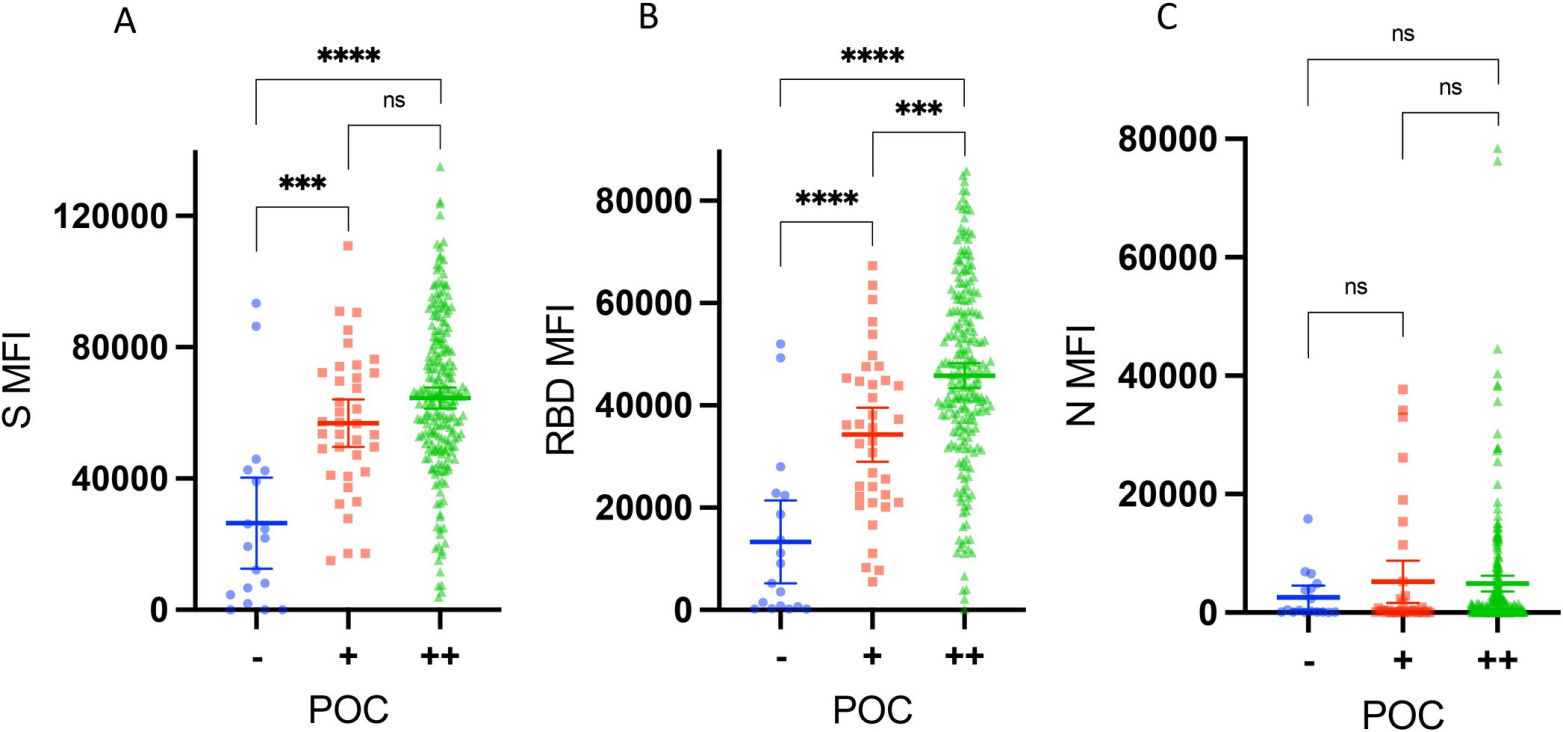
Antigen-specific median fluorescence intensity read out broken down by point-of-care test outcome. Comparing the point-of-care test outcome (POC) outcomes with antigen specific median fluorescence intensity (MFIs) revealed a correlation between a positive POC test and increasing MFI multiplex measurements for **(A)** S (ns, p = 0.06) and **(B)** RBD, but not for **(C)** N (ns, p = 0.19, p = 0.06, p = 0.86). MFI, median fluorescence intensity. N, Nucleocapsid. S, Spike. RBD, Receptor Binding Protein. POC, point-of-care test result. (-), POC negative. (+), POC positive (light band). (++), POC positive (dark band). Ns, non-significant (p≥0.05). *** = p<0.001. **** = p<0.0001.

The concordance between the POC test results and qualitative multiplex assay outcomes was high. Accordingly, 94.0% (267/284) of the outcomes aligned between the POC test and the three antigen RBD/S/N readouts (i.e.,17 outcomes did not match: 14 were positive by multiplex [RBD, S and N] but negative by POC, and 3 were negative by multiplex but positive by POC). Similarly, 94.4% (268/284) of the outcomes aligned between POC and the two antigen S/N analysis, 94.4% (268/284) of the outcomes aligned between POC and the two antigen RBD/N analysis, and 93.7% (266/284) of the outcomes aligned between POC and the two antigen RBD/S analysis. Comparing the POC outcomes with antigen specific MFIs revealed a correlation between a positive POC test and increasing MFI multiplex assay measurements for S and RBD, but not for N (see **Fig 2**), indicating that the POC test was reliably detecting the RBD/S antibodies measured by the multiplex assay.

**Fig 3:**
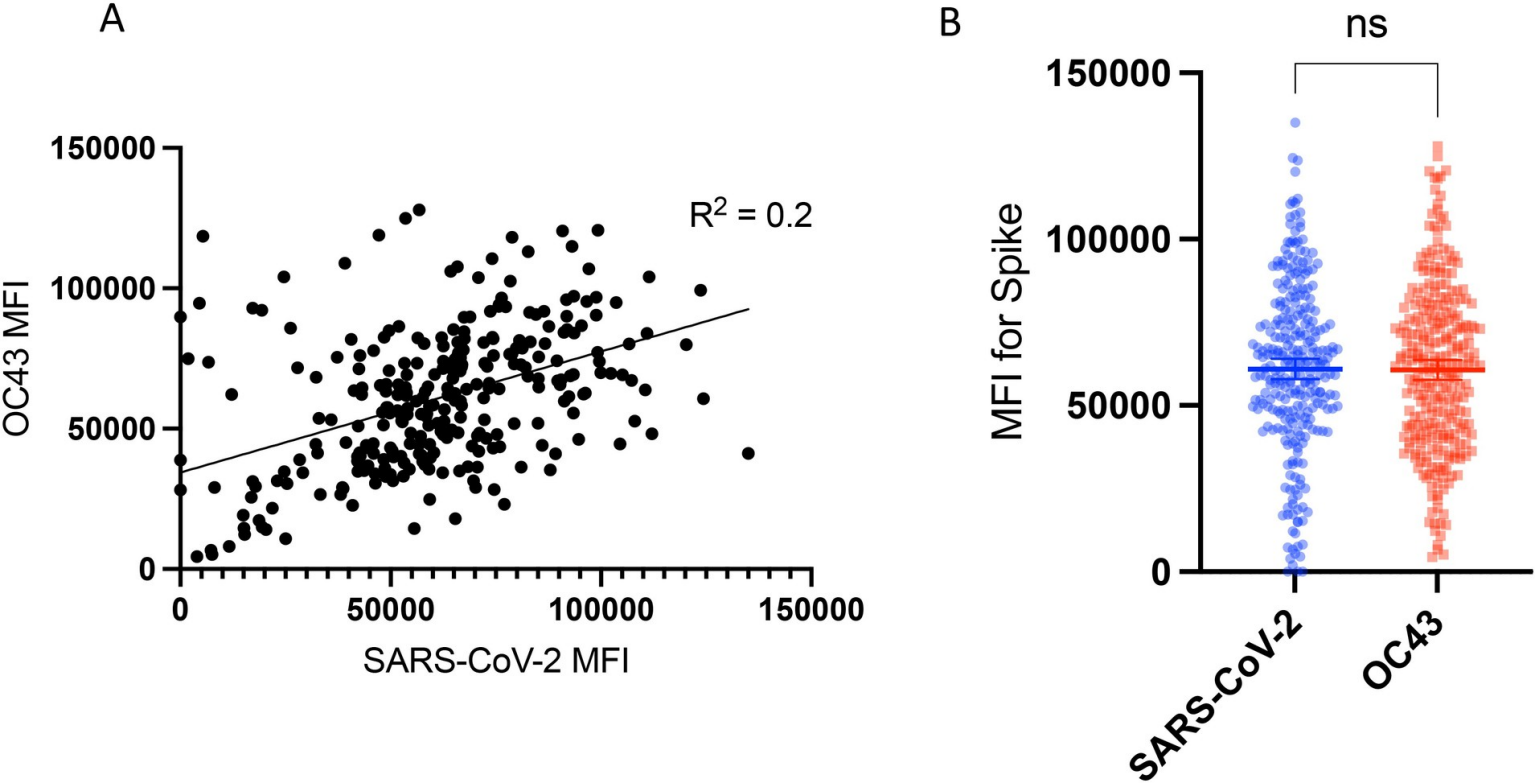
Comparison of SARS-CoV-2 and OC43 antibody measurements. **(A)** There was no/low correlation (R^2^ = 0.2) between SARS-CoV-2 and OC43 S-based MFI, both Spike [S]-based MFI. **(B)** Still, the paired antibody measurements for OC43 and SARS-CoV-2 were not significantly different by paired t test (p = 0.84). Hence, cross-reactivity between IgG S measurements for SARS-CoV-2 and OC43 could not be ruled out. MFI, median fluorescence intensity. Ns, non-significant (p≥0.05).

To analyze the potential cross-reactivity between SARS-CoV-2 and hCoVs, we evaluated IgG OC43, HKU1, 229E, and NL63 antibody levels in the blood samples. OC43 and 229E had the highest MFI outcomes, see **Fig 1**. Given that OC43 is closely related to SARS-CoV-2 (both β-CoV members) [14], we compared SARS-CoV-2 and OC43 antibody measurements (both S-based) and found the correlation to be low (R^2^ = 0.2), see **Fig 3**. Still, the antibody measurements for both were high, see **Fig 1,** and a paired t-test did not find the paired OC43 and SARS-CoV-2 measurements to be significantly different, see **Fig 3**. Hence, cross-reactivity between IgG S measurements for SARS-CoV-2 and OC43 could not be ruled out. We further analyzed the samples that were non-concordant between the multiplex analysis and the POC test for cross-reactivity (i.e., samples that were positive for the multiplex assay and negative for the POC test across all antigen combinations). The average SARS-CoV-2 S MFI of the non-concordant samples (n = 15) was lower compared to the concordant samples (n = 265), but the S-based OC43 measurements were not significantly higher (see **S3 Fig**). Hence, the sensitive and specific (**S2** and **S3 Tables** in S1 File) multiplex assay was more likely to pick up a positive SARS-CoV-2 sample compared to the POC test but it was not more likely to pick up OC43. Note that the statistical outcomes may be affected by the unequal sample sizes (15 vs 265).

While anti-SARS-CoV-2 IgA has been shown to wane faster than IgG, mucosal and blood-based IgA may provide protection from infections [7, 15–17]. Hence, we measured serum-based *IgA* seroprevalence which resulted in 87.2% ± 6.4% (95% CI) for RBD/S/N (3 antigens), 83.1% ± 6.7% (95% CI) for RBD/S (2 antigens), 84.8% ± 6.6% (95% CI) for S/N (2 antigens), 39.8% ± 12.2% (95% CI) for RBD/N (2 antigens), 62.7% ± 14.5% (95% CI) for RBD, 84.0% ± 6.7% (95% CI) for S, and 14.1% ± 25.5% (95% CI) for N, see **S3 Table** in S1 File. Comparing the antigen-specific outcomes in blood resulted in significantly lower MFIs for IgA compared to IgG for all antigens (p<0.0001; Welch’s t test), see **S4, S5,** and **S6 Figs**.

### Saliva SARS-CoV-2 antibodies

The saliva-based *IgG* seroprevalences and uncertainty range (approximating the 100% confidence interval) resulted in 100.0% (98.7–100.0) for RBD/S/N (3 antigens), 100.0% (98.7–100.0) for RBD/S (2 antigens), 96.0% (92.4–99.6) for S/N (2 antigens), 86.9% (81.6–96.4) for RBD/N (2 antigens), 86.9% (75.8–96.2) for RBD, 96.0% (92.4–99.6) for S, and 48.0% (48.0–99.7) for N, see **S4 Table** in S1 File. The SARS-CoV-2 seroprevalences from saliva and serum resulted in comparable outcomes for IgG (see **S2** and **S4 Tables** in S1 File), and the concordance between the qualitative positive/negative antibody results was high; 97.6% (279/286) of the results aligned for the IgG RBD/S/N (3 antigen) analysis (i.e., 7 participants had positive saliva samples but negative blood samples by IgG RBD/S/N multiplex analysis). Note that the concordance reflects the underlying high overall seroprevalence. A direct comparison of saliva and blood MFIs was not possible since saliva antibody measurements (MFI minus BSA, divided by total Ig, and multiplied by 1000) were transformed differently than blood/serum (MFI minus BSA) to account for variation in salivary flow rates and comparing the differently transformed MFIs between serum and saliva for each SARS-CoV-2 antigen did not identify a correlation, see **S7 Fig**.

The saliva-based *IgA* seroprevalences could not be calculated because the antigen-specific outcomes (transformed MFIs) between the study participant and alternative control samples overlapped significantly, see **S8 Fig**. Hence, no classification boundaries and therefore no percent seroprevalence could be established. However, comparing the antigen-specific measurements (MFI) in saliva resulted in significantly lower reads for IgA compared to IgG for the RBD and S antigen outcomes (p<0.0001; Welch’s t test), see **S9 and S10 Figs.** Whereas for the N-specific outcomes in saliva, there were less overall antibodies (e.g., lower transformed MFIs compared to RBD and S), and the IgA reads were significantly higher compared to IgG, see **S11 Fig**.

## Discussion

This study outlines an effective culturally sensitive recruitment method that overcame research study access barriers generally reported among US minority populations, enrolling 290 participants within four months [2, 18–20]. Among the mostly Latinx young to middle aged peri-urban MA study population, the majority reported being vaccinated (91.4%), which was confirmed by blood and saliva IgG antibody screening. According to the MA Department of Public Health, 86.9% of the MA population had received at least one dose of a COVID-19 vaccine by July 2022 [21] and 81.4% of the general US population had received at least one COVID-19 vaccine dose by May 2023 [22]. Hence, our diverse study population had a high vaccine uptake and did not reflect the reported vaccine hesitancy among minorities [19, 20, 23]. This may have been due to widely available COVID-19 vaccines in MA as vaccine availability and general ease of access has been cited as one of the main uptake barriers among marginalized groups [19, 20].

The percent of self-reported infections aligned with the N-based IgG seroprevalence outcomes in serum (N: 49.9%) and saliva (N: 48.0%). Hence, within two years and four months (the first COVID-19 case in MA was confirmed on Feb 1^st^, 2020 [24] and the study recruitment ended in July of 2022) about half our diverse study population had been exposed to SARS-CoV-2. While the percent of self-reported infections aligned with the N-based IgG serum and saliva seroprevalence results, we found that reporting a positive SARS-CoV-2 test was not necessarily linked to the presence of anti-N IgG antibodies (**Table 1**). This is likely due to the relatively short half-life of anti-N IgG antibodies. Others have found that anti-N antibodies start declining within one month post-positive PCR test with over half the study population testing seronegative within 6–7 months [25, 26]. Since our study was implemented 2+ years after the first local COVID-19 case and since antibody levels may range across individuals, it is likely that the antibody levels had dropped below the detection limits by the time we collected and screened the blood samples of these individuals. Similarly, we found that participants who did not report a confirmatory test were positive for anti-N IgG antibodies. Given that 4% to 41% of SARS-CoV-2 infections may be asymptomatic [27], it is likely that these individuals may have been infected but were not aware or did not seek testing.

The reported average severity scores were higher for infections (5.4) as compared to vaccination (4.1 and 3.6 for baseline doses and booster, respectively) and the overall number of vaccine-associated hospitalizations were lower (n = 6) compared to infection-associated hospitalizations (n = 11). Additionally, 26.5% of individuals who reported past infections had not fully recovered and 14.1% reported long COVID-19 symptoms. Among those who experienced lingering COVID-19 symptoms, most were female (78.1%), Latinx (87.5%, n = 28), and from mixed/mestizo racial background (59.4%, n = 19), while the average age was 48.6 years. Hence, indicating that long-term COVID-19 symptoms were prevalent among our community-based study population. Our results were consistent with previous studies that reported being 50+ years old and being from disadvantaged ethnic and socioeconomic groups as a risk factor, although comorbidities did not correlate with lack of COVID-19 symptom resolution, which could have been due to our small sample size of participants with lingering COVID-19 symptoms [28].

Finding high concordance (93.7%) between the POC results and the RBD/S (2 antigen excluding N) analysis for IgG outcomes in blood and a correlation between a positive POC test and increasing MFI multiplex measurements for IgG S and RBD (but not for N) in blood indicated that the RBD and S measurements (more so than N) were driving the overlapping results between the POC and multiplex outcome in our study population. Our and other studies have found N-based antibody levels to be lower and more variable (i.e., shorter half-life than S/RBD) [29, 30]. Hence, while we found that the POC test was an easy to use and reliable IgG vaccine-induced antibody measurement tool, the multiplex assay was more likely to pick up a positive sample and is more appropriate for serosurveys targeting and differentiating between infection- and vaccine-induced antibodies.

While the majority of SARS-CoV-2 serosurveys do not account for cross-reactivity between SARS-CoV-2 and hCoV measurements, we found that readout overlap for S between SARS-CoV-2 and OC43 could not be ruled out in our setting, indicating the need for further scrutiny in future serosurveys. This is particularly true because most individuals are thought to seroconvert for hCoVs during childhood [31–33] and variation in hCoV infection history has been proposed to induce protective immunity from COVID-19 [33–35].

In terms of methods, a current major challenge of serological analytics is (i) the application of validated across-plate normalization methods to pool outcomes from a large sample size, and (ii) the determination of threshold values to reliably convert quantitative outcomes (MFIs) into qualitative results (positive/negative) [9]. We therefore validated a weighted-standard curve across-plate normalization method and two classification boundary methods for optimal qualitative serological assessments (one based on pre-defined positive and negative controls and one based on an alternate control group), across two isotypes (IgG and IgA) and two sample types (serum and saliva). As shown in the results, applying our methods resulted in the alignment of survey answers, POC results, and IgG-based serological outcomes with high classification accuracy, sensitivity and specificity in serum (**S2** and **S3 Tables** in S1 File) and saliva for almost all antigen combinations.

Specific to saliva-based serological analytics, (i) variation in salivary flow rate due to changes in circadian rhythm, stress, and sample collection method [36], and (ii) across sample variation in isotype specific-outcomes (IgA and IgG) due to inherent biological mechanisms (i.e., antibody source and half-life) are a major challenge [37–39]. Further, it is problematic to pool saliva-based serological outcomes across studies and identify appropriate controls since different saliva collection methods influence the composition and quality of the collected samples [40]. Here, we compared multiplex-based anti-SARS-CoV-2 IgG and IgA antibody measurements in matched serum and saliva samples. Our IgG-based serological outcomes in serum and saliva aligned, supporting the use of saliva as a less-invasive and accessible sample particularly among hesitant research participants. However, we found significant differences in antigen-specific IgA vs. IgG antibody levels, similarly to previous reports [7, 41]. This was likely because (i) the half-life of IgA is shorter compared to IgG, and (ii) mucosal and systemic IgA production are not synchronized [7, 37, 39, 41]. Further, the antigen-specific outcomes (MFI minus BSA for serum and transformed MFI for saliva) between serum and saliva did not correlate, even though the antigen-specific IgG seroprevalences aligned, underlining the importance of including appropriate controls and threshold calculation methods for final outcome comparisons.

The main limitation of our study was the restricted sample size. Hence, while our results generally align with previously published data, the statistical analyses and comparisons among infected individuals need to be confirmed among larger diverse populations. Further, most of our population carried anti-SAR-CoV-2 antibodies so subsequent statistical comparisons were restricted by the lack of negative outcomes.

In summary, this study successfully engaged marginalized MA communities and evaluated the impact of COVID-19 and vaccine uptake by implementing culturally sensitive recruitment methods and by giving appropriate study participant compensation in the form of immediate antibody results and adequate time and travel reimbursements.

We found a high vaccine uptake, and that about half of the participants were infected with SARS-CoV-2 within 2+ years of the beginning of the pandemic. We found that lingering COVID-19 symptoms were prevalent and impacted mostly middle-aged female Latinas, indicating continued need for public health attention despite high COVID-19 vaccine uptake. By comparison of matched blood and saliva samples, we found that saliva served as a reliable non-invasive alternative for IgG but not IgA antibody measurements, and we successfully adapted across plate normalization and classification boundary methods for optimal qualitative serological assessments. We also found that the bead-based multiplex assay had high overall sensitivity and specificity for blood samples and was more likely to pick up a positive sample than the POC. Overall, the bead-based multiplex assay was better suited for serosurveys targeting infection- and vaccine-induced antibodies compared to the less labor-intensive POC test and that hCoV cross-reactivity should be evaluated for reliable SARS-CoV-2 serosurvey results.

## Supporting information

S1 Fig Representative graphic of a three-dimensional classification boundary. The graphic of a three-dimensional (3D) classification boundary graphic was based on training data covering anti-RBD, -S, and -N antibodies from confirmed positive and negative samples. S, Spike. RBD, Receptor Binding Protein. N, Nucleocapsid.
(TIFF)

S2 Fig Serum IgG outcome distribution in linear scale. Linear scale dot plot with means and 95% confidence intervals (CI) of antigen-specific antibody measurements (MFI minus BSA and normalized across plates) for serum IgG SARS-CoV-2 and variants, along with human endemic coronaviruses OC43, HKU1, NL63, 229E. MFI, median fluorescence intensity. N, Nucleocapsid. S, Spike. RBD, Receptor Binding Protein. hCoV, human endemic coronaviruses. WT, wild-type (Wuhan).
(TIFF)

S3 Fig Analysis of samples that were non-concordant between the multiplex analysis and the point-of care test. Comparison of samples that were positive for the multiplex assay and negative for the point-of care test (POC) test across all antigen combinations). (**A**) The average SARS-CoV-2 spike (S) median fluorescence intensity (MFI) of the non-concordant samples (n = 15) was lower compared to the concordant samples (n = 265, p = 0.0005). (**B**) The S-based OC43 measurements of the non-concordant samples were not significantly higher than the concordant samples (p = 0.12). Hence, while the multiplex assay exhibited high sensitivity and specificity for blood samples (**S2** and **S3 Tables** in S1 File) and was more likely to pick up a positive SARS-CoV-2 sample compared to the POC test, it was not more likely to pick up a positive OC43 sample among the non-concordant samples. The statistical outcomes may be affected by the unequal sample sizes (15 vs 265). MFI, median fluorescence intensity. S, Spike. Ns, non-significant (p>0.05). Non-conc., non-concordant samples (multiplex assay vs. POC test). Conc., concordant samples (sample that had the same qualitative outcome both with the multiplex assay and POC test). POC, point-of-care test. *** = p<0.001, Welch’s t-test.
(TIFF)

S4 Fig Comparison of receptor binding protein-specific serum IgG and IgA outcomes. Comparison of receptor binding protein (RBD)-specific serum IgG and IgA outcomes as median fluorescence intensity (MFI, mean and standard deviations) among the study participants (mean and standard deviations). **** = p<0.0001, Welch’s t-test. MFI, median fluorescence intensity. RBD, Receptor Binding Protein.
(TIFF)

S5 Fig Comparison of spike-specific serum IgG and IgA outcomes. Comparison of spike (S) protein-specific serum IgG and IgA outcomes (MFIs) among the study participants (mean and standard deviations). **** = p<0.0001, Welch’s t-test. S, Spike Protein.
(TIFF)

S6 Fig Comparison of nucleocapsid-specific serum IgG and IgA outcomes. Comparison of nucleocapsid (N) protein-specific serum IgG and IgA outcomes (MFIs) among the study participants (mean and standard deviations). **** = p<0.0001, Welch’s t-test. N, Nucleocapsid Protein.
(TIFF)

S7 Fig Comparison of serum- and saliva-based serological outcomes. Line up of saliva versus serum comparisons of antigen-specific outcomes (MFI minus BSA for serum and transformed MFI for saliva [antigen- and isotype-specific MFI minus BSA, divided by total Ig, multiplied by 1000]), for anti-SARS-CoV-2 receptor binding domain (RBD; **A, B**), spike (S; **C, D**), and nucleocapsid (N; **E, F**) IgG (left column) and IgA (right column) antibody measurements. The outcomes between serum and saliva did not correlate for any antigen or isotype combination.
(TIF)

S8 Fig Comparison of serological outcomes from study versus control samples. Comparison of study and control population by line up of saliva-based antigen-specific transformed MFI (antigen- and isotype-specific MFI minus BSA, divided by total Ig, and multiplied by 1000) for IgG (left column) and IgA (right column). For saliva IgG, the control sample population (sample collection method-matched samples from Kenya) always clusters in low MFI area and separate well from the study sample population (**A, C, E**), whereas for IgA the outcomes/MFIs from the control sample population overlap significantly with the study sample population for at least one antigen (**B, D, F**) and score higher maximum MFIs for RBD-specific outcomes (**B, D**). Hence, no saliva IgA percent seroprevalences could be calculated for the study samples based on these controls. S, Spike. RBD, Receptor Binding Protein. N, Nucleocapsid.
(TIF)

S9 Fig Comparison of receptor binding protein-specific saliva IgG and IgA serological outcomes. Comparison of RBD-specific saliva IgG and IgA outcomes (transformed MFI = [raw MFI/total Ig]*1000) among the study participants (mean and standard deviations). **** = p<0.0001, Welch’s t-test. RBD, Receptor Binding Protein.
(TIFF)

S10 Fig Comparison of spike protein-specific saliva IgG and IgA serological outcomes. Comparison of S-specific saliva IgG and IgA outcomes (transformed MFI = [raw MFI/total Ig]*1000) among the study participants (mean and standard deviations). **** = p<0.0001, Welch’s t-test. S, Spike.
(TIFF)

S11 Fig Comparison of nucleocapsid protein-specific saliva IgG and IgA serological outcomes. Comparison of nucleocapsid (N)-specific saliva IgG and IgA outcomes (transformed MFI = [raw MFI/total Ig]*1000) among the study participants (mean and standard deviations). **** = p<0.0001, Welch’s t-test. For N-specific outcomes in saliva, the IgA reads are higher compared to IgG. Whereas for RBD and S, the IgG reads in saliva are higher. Overall, the N-specific saliva IgG and IgA outcomes (transformed MFI) are lower than for RBD and S (i.e., lower overall MFI, see y-axis comparison between S8, S9, and S10 Figs). N, Nucleocapsid.
(TIFF)

S1 File This is a template of the data collection survey utilized for this study. (PDF)

S2 File This document contains supplemental information on the study and associated methods as referenced throughout the manuscript. (PDF)

S1 Dataset This document contains the raw data utilized for the study analysis, covering both the survey results and serological outcomes. (XLSX)

## References

[1] Kullar R, Marcelin JR, Swartz TH, Piggott DA, Macias Gil R, Mathew TA, et al. Racial Disparity of Coronavirus Disease 2019 in African American Communities. J Infect Dis. 2020;222(6):890–3. Epub 2020/07/01. doi: 10.1093/infdis/jiaa372 ; PubMed Central PMCID: PMC7337812. 32599614 PMC7337812

[2] Mackey K, Ayers CK, Kondo KK, Saha S, Advani SM, Young S, et al. Racial and Ethnic Disparities in COVID-19-Related Infections, Hospitalizations, and Deaths: A Systematic Review. Ann Intern Med. 2021;174(3):362–73. Epub 2020/12/01. doi: 10.7326/M20-6306 ; PubMed Central PMCID: PMC7772883 at www.acponline.org/authors/icmje/ConflictOfInterestForms.do?msNum=M20-6306. 33253040 PMC7772883

[3] Larremore DB, Fosdick BK, Bubar KM, Zhang S, Kissler SM, Metcalf CJE, et al. Estimating SARS-CoV-2 seroprevalence and epidemiological parameters with uncertainty from serological surveys. Elife. 2021;10. Epub 2021/03/06. doi: 10.7554/eLife.64206 ; PubMed Central PMCID: PMC7979159. 33666169 PMC7979159

[4] Farnsworth CW, Anderson NW. SARS-CoV-2 Serology: Much Hype, Little Data. Clin Chem. 2020;66(7):875–7. Epub 2020/04/29. doi: 10.1093/clinchem/hvaa107 ; PubMed Central PMCID: PMC7197624. 32343775 PMC7197624

[5] CDC. Interim Guidelines for COVID-19 Antibody Testing 2022 [July 1 2022]. Available from: https://www.cdc.gov/coronavirus/2019-ncov/lab/resources/antibody-tests-guidelines.html.

[6] Jones JM, Stone M, Sulaeman H, Fink RV, Dave H, Levy ME, et al. Estimated US Infection- and Vaccine-Induced SARS-CoV-2 Seroprevalence Based on Blood Donations, July 2020-May 2021. JAMA. 2021;326(14):1400–9. Epub 2021/09/03. doi: 10.1001/jama.2021.15161 ; PubMed Central PMCID: PMC8414359. 34473201 PMC8414359

[7] Isho B, Abe KT, Zuo M, Jamal AJ, Rathod B, Wang JH, et al. Persistence of serum and saliva antibody responses to SARS-CoV-2 spike antigens in COVID-19 patients. Sci Immunol. 2020;5(52). Epub 2020/10/10. doi: 10.1126/sciimmunol.abe5511 ; PubMed Central PMCID: PMC8050884. 33033173 PMC8050884

[8] Pickering JW, Larson MT, Martins TB, Copple SS, Hill HR. Elimination of false-positive results in a luminex assay for pneumococcal antibodies. Clin Vaccine Immunol. 2010;17(1):185–9. Epub 2009/11/20. doi: 10.1128/CVI.00329-09 ; PubMed Central PMCID: PMC2812081. 19923569 PMC2812081

[9] Binder RA, Fujimori GF, Forconi CS, Reed GW, Silva LS, Lakshmi PS, et al. SARS-CoV-2 Serosurveys: How antigen, isotype and threshold choices affect the outcome. J Infect Dis. 2022. Epub 2022/11/01. doi: 10.1093/infdis/jiac431 . 36314635 PMC9891417

[10] Patrone P.N.; Binder R.A.; Forconi C.S.; Moormann A.M.; Kearsley A.J. Minimal Assumptions for Optimal Serology Classification: Theory and Implications for Multidimensional Settings and Impure Training Data. arXiv. 2023. 10.48550/arXiv.2309.00645.

[11] Wiemers EE, Abrahams S, AlFakhri M, Hotz VJ, Schoeni RF, Seltzer JA. Disparities in vulnerability to complications from COVID-19 arising from disparities in preexisting conditions in the United States. Res Soc Stratif Mobil. 2020;69:100553. Epub 20200907. doi: 10.1016/j.rssm.2020.100553 ; PubMed Central PMCID: PMC7476505. 32921870 PMC7476505

[12] Tai DBG, Shah A, Doubeni CA, Sia IG, Wieland ML. The Disproportionate Impact of COVID-19 on Racial and Ethnic Minorities in the United States. Clin Infect Dis. 2021;72(4):703–6. doi: 10.1093/cid/ciaa815 ; PubMed Central PMCID: PMC7337626. 32562416 PMC7337626

[13] Richardson S, Hirsch JS, Narasimhan M, Crawford JM, McGinn T, Davidson KW, et al. Presenting Characteristics, Comorbidities, and Outcomes Among 5700 Patients Hospitalized With COVID-19 in the New York City Area. JAMA. 2020;323(20):2052–9. doi: 10.1001/jama.2020.6775 ; PubMed Central PMCID: PMC7177629. 32320003 PMC7177629

[14] Ding X. Liu JQL, and Fung To S. Human Coronavirus-229E, -OC43, -NL63, and -HKU1 (Coronaviridae). Encyclopedia of Virology 2021.

[15] Takamatsu Y, Omata K, Shimizu Y, Kinoshita-Iwamoto N, Terada M, Suzuki T, et al. SARS-CoV-2-neutralizing humoral IgA response occurs earlier but modest and diminishes faster compared to IgG response. bioRxiv. 2022. Epub 2022/06/16. doi: 10.1101/2022.06.09.495422 ; PubMed Central PMCID: PMC9196114 exist. 36219096 PMC9769934

[16] Vata A, Anita A, Manciuc CD, Savuta G, Luca CM, Rosu FM, et al. Clinical significance of early IgA anti-SARS-CoV-2 antibody detection in patients from a Romanian referral COVID-19 hospital. Exp Ther Med. 2022;23(6):391. Epub 2022/05/03. doi: 10.3892/etm.2022.11318 ; PubMed Central PMCID: PMC9019744. 35495593 PMC9019744

[17] Montague BT, Wipperman MF, Chio E, Crow R, Hooper AT, O’Brien MP, Simoes EAF. Elevated serum IgA following vaccination against SARS-CoV-2 in a cohort of high-risk first responders. Sci Rep. 2022;12(1):14932. Epub 2022/09/03. doi: 10.1038/s41598-022-19095-7 ; PubMed Central PMCID: PMC9437396 investigator-initiated study, with funding from Regeneron Pharmaceuticals to Eric Simoes PI and Brian Montague Co-Investigator Grant No. 0000-COV-CES-2053. Final decisions regarding study design, implementation, data analysis and manuscript preparation were made by the principal investigator and co-investigator. Coauthors affiliated with Regeneron Pharmaceuticals provided collaborative assistance with coordination of laboratory testing, data review and analysis, and review of the final manuscript. M.F.W., A.T.H., S.C.H., F.E., L.H., S.L., J.D.H. and M.P.O. are current employees and stockholders of Regeneron Pharmaceuticals. A.T.H. is a prior employee and stockholder for Pfizer, Inc. All authors declare no other competing interests. 36056118 PMC9437396

[18] Paskett ED, Reeves KW, McLaughlin JM, Katz ML, McAlearney AS, Ruffin MT, et al. Recruitment of minority and underserved populations in the United States: the Centers for Population Health and Health Disparities experience. Contemp Clin Trials. 2008;29(6):847–61. Epub 2008/08/30. doi: 10.1016/j.cct.2008.07.006 ; PubMed Central PMCID: PMC2642621. 18721901 PMC2642621

[19] Corbie-Smith G. Vaccine Hesitancy Is a Scapegoat for Structural Racism. JAMA Health Forum. 2021;2(3):e210434. Epub 2021/03/01. doi: 10.1001/jamahealthforum.2021.0434 . 36218456

[20] Quinn SC, Andrasik MP. Addressing Vaccine Hesitancy in BIPOC Communities—Toward Trustworthiness, Partnership, and Reciprocity. N Engl J Med. 2021;385(2):97–100. Epub 2021/04/01. doi: 10.1056/NEJMp2103104 . 33789007

[21] Daily COVID-19 Vaccine Report [Internet]. 2022. Available from: chrome-extension://efaidnbmnnnibpcajpcglclefindmkaj/https://www.mass.gov/doc/daily-covid-19-vaccine-report-july-1-2022/download.

[22] COVID Data Tracker [Internet]. 2023. Available from: https://covid.cdc.gov/covid-data-tracker/#vaccinations_vacc-people-booster-percent-pop5.

[23] Fisher C, Bragard E, Madhivanan P. COVID-19 Vaccine Hesitancy among Economically Marginalized Hispanic Parents of Children under Five Years in the United States. Vaccines (Basel). 2023;11(3). Epub 2023/03/31. doi: 10.3390/vaccines11030599 ; PubMed Central PMCID: PMC10052092. 36992183 PMC10052092

[24] Burke M. Coronavirus case in Boston is 1st in Massachusetts; 8th in the U.S. NBC News. 2020.

[25] Van Elslande J, Gruwier L, Godderis L, Vermeersch P. Estimated Half-Life of SARS-CoV-2 Anti-Spike Antibodies More Than Double the Half-Life of Anti-nucleocapsid Antibodies in Healthcare Workers. Clin Infect Dis. 2021;73(12):2366–8. doi: 10.1093/cid/ciab219 ; PubMed Central PMCID: PMC7989510. 33693643 PMC7989510

[26] Lumley SF, Wei J, O’Donnell D, Stoesser NE, Matthews PC, Howarth A, et al. The Duration, Dynamics, and Determinants of Severe Acute Respiratory Syndrome Coronavirus 2 (SARS-CoV-2) Antibody Responses in Individual Healthcare Workers. Clin Infect Dis. 2021;73(3):e699–e709. doi: 10.1093/cid/ciab004 ; PubMed Central PMCID: PMC7929225. 33400782 PMC7929225

[27] Byambasuren O, Cardona M, Bell K, Clark J, McLaws ML, Glasziou P. Estimating the extent of asymptomatic COVID-19 and its potential for community transmission: Systematic review and meta-analysis. J Assoc Med Microbiol Infect Dis Can. 2020;5(4):223–34. Epub 20201231. doi: 10.3138/jammi-2020-0030 ; PubMed Central PMCID: PMC9602871. 36340059 PMC9602871

[28] Turner S, Khan MA, Putrino D, Woodcock A, Kell DB, Pretorius E. Long COVID: pathophysiological factors and abnormalities of coagulation. Trends Endocrinol Metab. 2023;34(6):321–44. Epub 2023/04/21. doi: 10.1016/j.tem.2023.03.002 ; PubMed Central PMCID: PMC10113134. 37080828 PMC10113134

[29] Whitcombe AL, McGregor R, Craigie A, James A, Charlewood R, Lorenz N, et al. Comprehensive analysis of SARS-CoV-2 antibody dynamics in New Zealand. Clin Transl Immunology. 2021;10(3):e1261. Epub 2021/03/23. doi: 10.1002/cti2.1261 ; PubMed Central PMCID: PMC7955949. 33747511 PMC7955949

[30] Vogelzang EH, Loeff FC, Derksen NIL, Kruithof S, Ooijevaar-de Heer P, van Mierlo G, et al. Development of a SARS-CoV-2 Total Antibody Assay and the Dynamics of Antibody Response over Time in Hospitalized and Nonhospitalized Patients with COVID-19. J Immunol. 2020;205(12):3491–9. Epub 2020/11/01. doi: 10.4049/jimmunol.2000767 . 33127820

[31] Dijkman R, Jebbink MF, Gaunt E, Rossen JW, Templeton KE, Kuijpers TW, van der Hoek L. The dominance of human coronavirus OC43 and NL63 infections in infants. J Clin Virol. 2012;53(2):135–9. Epub 2011/12/23. doi: 10.1016/j.jcv.2011.11.011 ; PubMed Central PMCID: PMC7108278. 22188723 PMC7108278

[32] Corman VM, Muth D, Niemeyer D, Drosten C. Hosts and Sources of Endemic Human Coronaviruses. Adv Virus Res. 2018;100:163–88. Epub 2018/03/20. doi: 10.1016/bs.aivir.2018.01.001 ; PubMed Central PMCID: PMC7112090. 29551135 PMC7112090

[33] Li P, Ikram A, Peppelenbosch MP, Ma Z, Pan Q. Systematically Mapping Clinical Features of Infections With Classical Endemic Human Coronaviruses. Clin Infect Dis. 2021;73(3):554–5. Epub 2020/09/15. doi: 10.1093/cid/ciaa1386 ; PubMed Central PMCID: PMC7543303. 32926168 PMC7543303

[34] Sagar M, Reifler K, Rossi M, Miller NS, Sinha P, White LF, et al. Recent endemic coronavirus infection is associated with less-severe COVID-19. J Clin Invest. 2021;131(1). Epub 2020/10/01. doi: 10.1172/JCI143380 ; PubMed Central PMCID: PMC7773342. 32997649 PMC7773342

[35] Tsinda EK, Mmbando GS. Recent updates on the possible reasons for the low incidence and morbidity of COVID-19 cases in Africa. Bull Natl Res Cent. 2021;45(1):133. Epub 2021/08/03. doi: 10.1186/s42269-021-00589-9 ; PubMed Central PMCID: PMC8300982. 34335014 PMC8300982

[36] Sano K, Bhavsar D, Singh G, Floda D, Srivastava K, Gleason C, et al. SARS-CoV-2 vaccination induces mucosal antibody responses in previously infected individuals. Nat Commun. 2022;13(1):5135. Epub 2022/09/02. doi: 10.1038/s41467-022-32389-8 ; PubMed Central PMCID: PMC9435409 relating to SARS-CoV-2 serological assays and NDV-based SARS-CoV-2 vaccines which list F.K. as co-inventor. V.S. is also listed on the serological assay patent application as co-inventor. Mount Sinai has spun out a company, Kantaro, to market serological tests for SARS-CoV-2. F.K. has consulted for Merck and Pfizer (before 2020) and is currently consulting for Pfizer, Seqirus, Third Rock Ventures, and Avimex. The Krammer laboratory is also collaborating with Pfizer on animal models of SARS-CoV-2. The remaining authors declare no competing interests. 36050304 PMC9435409

[37] van Tetering G, Evers M, Chan C, Stip M, Leusen J. Fc Engineering Strategies to Advance IgA Antibodies as Therapeutic Agents. Antibodies (Basel). 2020;9(4). Epub 2020/12/19. doi: 10.3390/antib9040070 ; PubMed Central PMCID: PMC7768499. 33333967 PMC7768499

[38] Brandtzaeg P. Secretory immunity with special reference to the oral cavity. J Oral Microbiol. 2013;5. Epub 2013/03/15. doi: 10.3402/jom.v5i0.20401 ; PubMed Central PMCID: PMC3595421. 23487566 PMC3595421

[39] Ceron JJ, Lamy E, Martinez-Subiela S, Lopez-Jornet P, Capela ESF, Eckersall PD, et al. Use of Saliva for Diagnosis and Monitoring the SARS-CoV-2: A General Perspective. J Clin Med. 2020;9(5). Epub 2020/05/21. doi: 10.3390/jcm9051491 ; PubMed Central PMCID: PMC7290439. 32429101 PMC7290439

[40] Laxton CS, Peno C, Hahn AM, Allicock OM, Perniciaro S, Wyllie AL. The potential of saliva as an accessible and sensitive sample type for the detection of respiratory pathogens and host immunity. Lancet Microbe. 2023;4(10):e837–e50. Epub 2023/07/30. doi: 10.1016/S2666-5247(23)00135-0 . 37516121 PMC12614176

[41] Sterlin D, Mathian A, Miyara M, Mohr A, Anna F, Claer L, et al. IgA dominates the early neutralizing antibody response to SARS-CoV-2. Sci Transl Med. 2021;13(577). Epub 2020/12/09. doi: 10.1126/scitranslmed.abd2223 ; PubMed Central PMCID: PMC7857408. 33288662 PMC7857408

