# Supplementary figures and images for: Minding the margins: Evaluating the impact of COVID-19 among Latinx and Black communities with optimal qualitative serological assessment tools
Source: PLoS One. 2024 Jul 25;19(7):e0307568. doi: 10.1371/journal.pone.0307568 (PMC11271856; doi:10.1371/journal.pone.0307568)

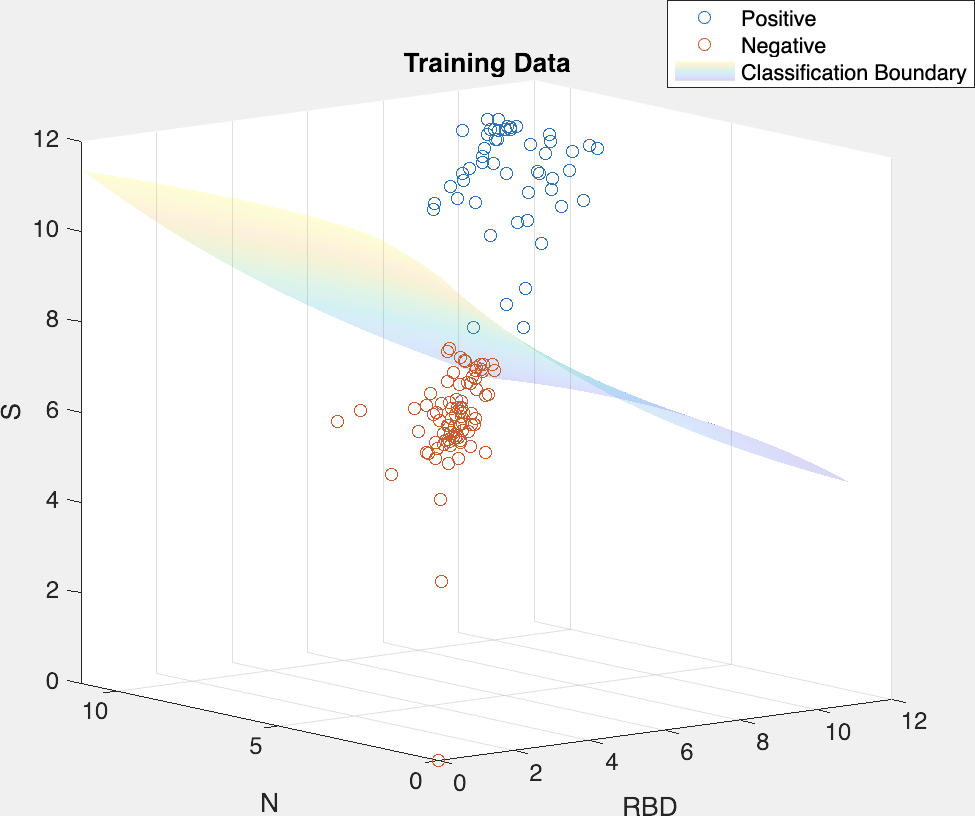

Supplement: S1 Fig — The graphic of a three-dimensional (3D) classification boundary graphic was based on training data covering anti-RBD, -S, and -N antibodies from confirmed positive and negative samples. S, Spike. RBD, Receptor Binding Protein. N, Nucleocapsid. (TIFF) [file pone.0307568.s001.tiff]

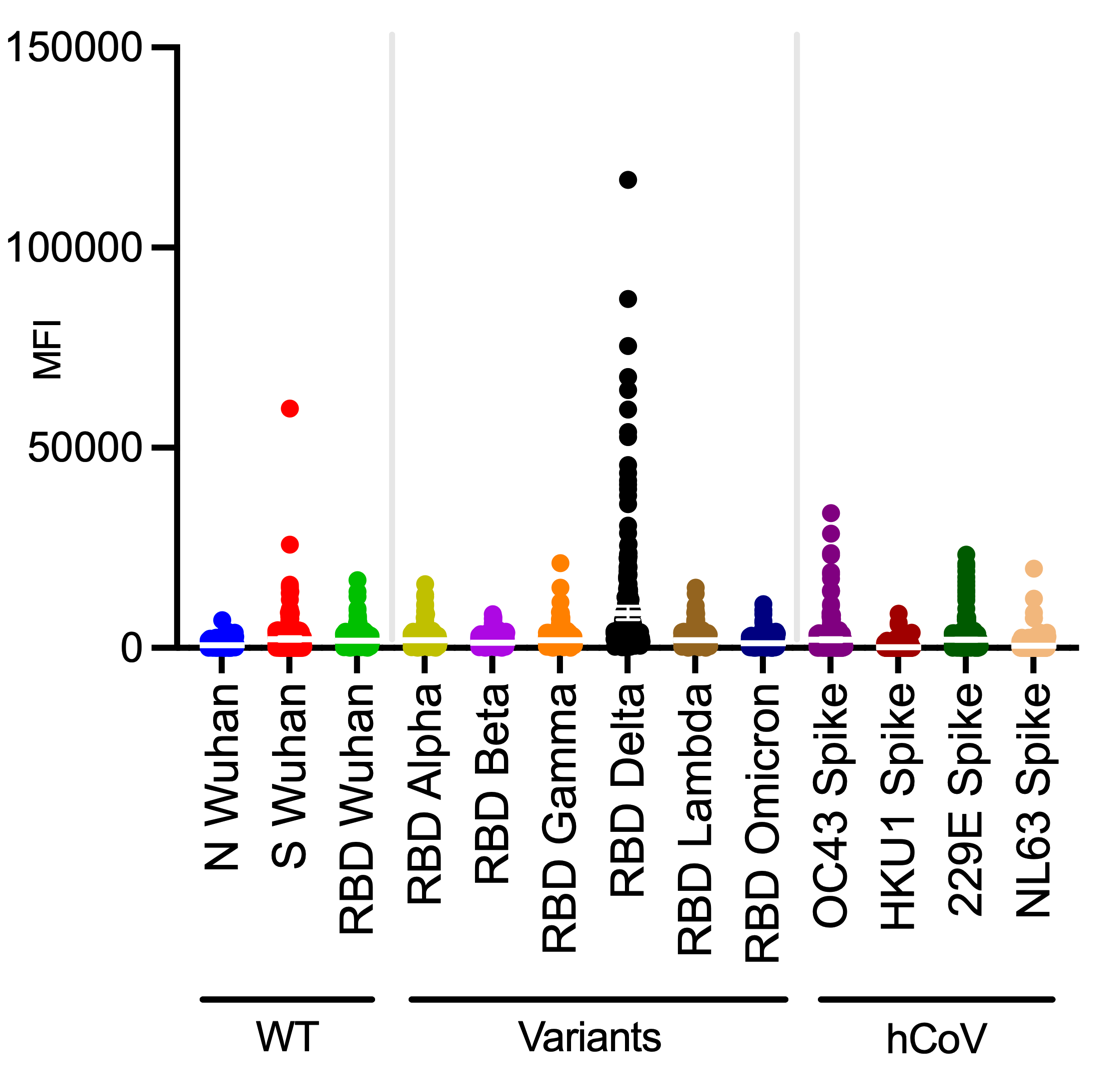

Supplement: S2 Fig — Linear scale dot plot with means and 95% confidence intervals (CI) of antigen-specific antibody measurements (MFI minus BSA and normalized across plates) for serum IgG SARS-CoV-2 and variants, along with human endemic coronaviruses OC43, HKU1, NL63, 229E. MFI, median fluorescence intensity. N, Nucleocapsid. S, Spike. RBD, Receptor Binding Protein. hCoV, human endemic coronaviruses. WT, wild-type (Wuhan). (TIFF) [file pone.0307568.s002.tiff]

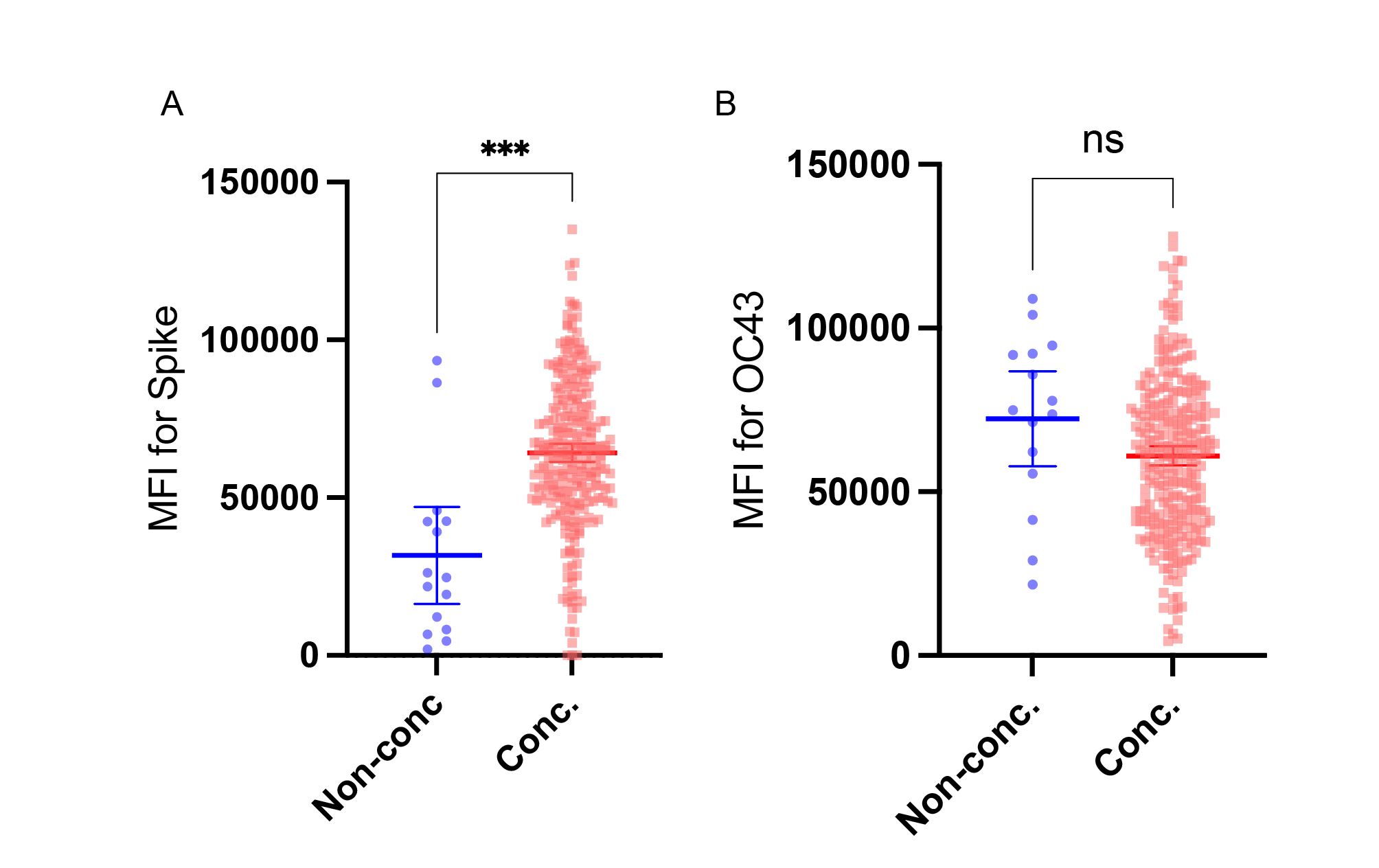

Supplement: S3 Fig — Comparison of samples that were positive for the multiplex assay and negative for the point-of care test (POC) test across all antigen combinations). (A) The average SARS-CoV-2 spike (S) median fluorescence intensity (MFI) of the non-concordant samples (n = 15) was lower compared to the concordant samples (n = 265, p = 0.0005). (B) The S-based OC43 measurements of the non-concordant samples were not significantly higher than the concordant samples (p = 0.12). Hence, while the multiplex assay exhibited high sensitivity and specificity for blood samples (S2 and S3 Tables in S1 File) and was more likely to pick up a positive SARS-CoV-2 sample compared to the POC test, it was not more likely to pick up a positive OC43 sample among the non-concordant samples. The statistical outcomes may be affected by the unequal sample sizes (15 vs 265). MFI, median fluorescence intensity. S, Spike. Ns, non-significant (p>0.05). Non-conc., non-concordant samples (multiplex assay vs. POC test). Conc., concordant samples (sample that had the same qualitative outcome both with the multiplex assay and POC test). POC, point-of-care test. *** = p<0.001, Welch’s t-test. (TIFF) [file pone.0307568.s003.tiff]

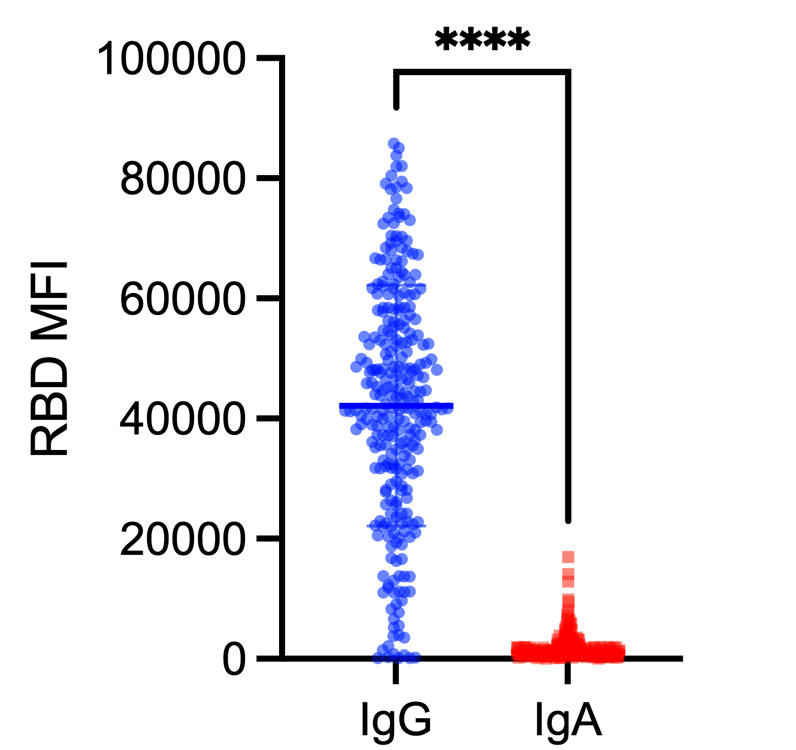

Supplement: S4 Fig — Comparison of receptor binding protein (RBD)-specific serum IgG and IgA outcomes as median fluorescence intensity (MFI, mean and standard deviations) among the study participants (mean and standard deviations). **** = p<0.0001, Welch’s t-test. MFI, median fluorescence intensity. RBD, Receptor Binding Protein. (TIFF) [file pone.0307568.s004.tiff]

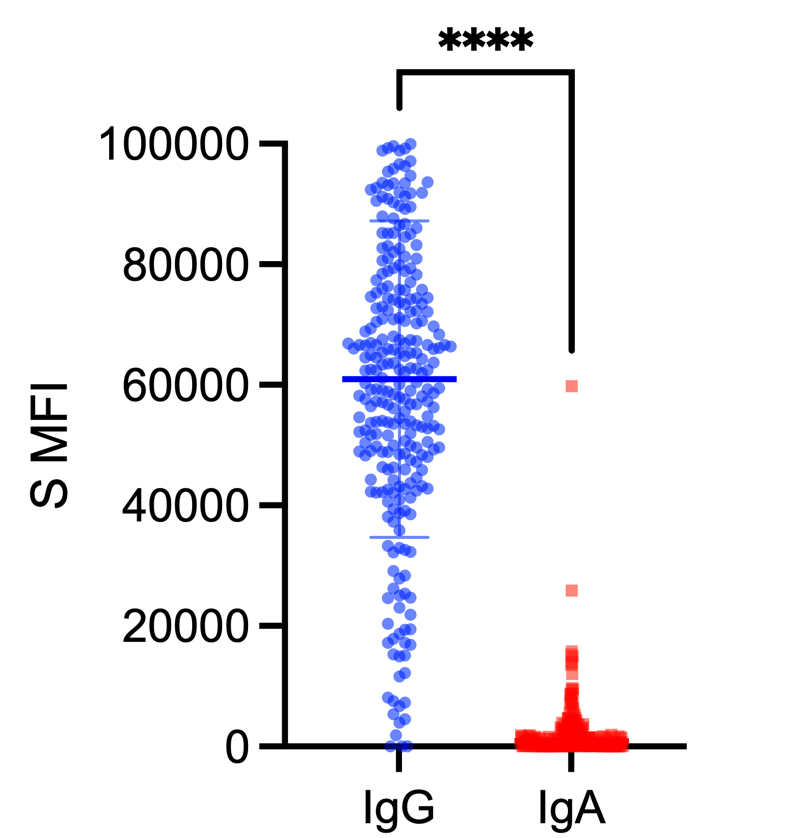

Supplement: S5 Fig — Comparison of spike (S) protein-specific serum IgG and IgA outcomes (MFIs) among the study participants (mean and standard deviations). **** = p<0.0001, Welch’s t-test. S, Spike Protein. (TIFF) [file pone.0307568.s005.tiff]

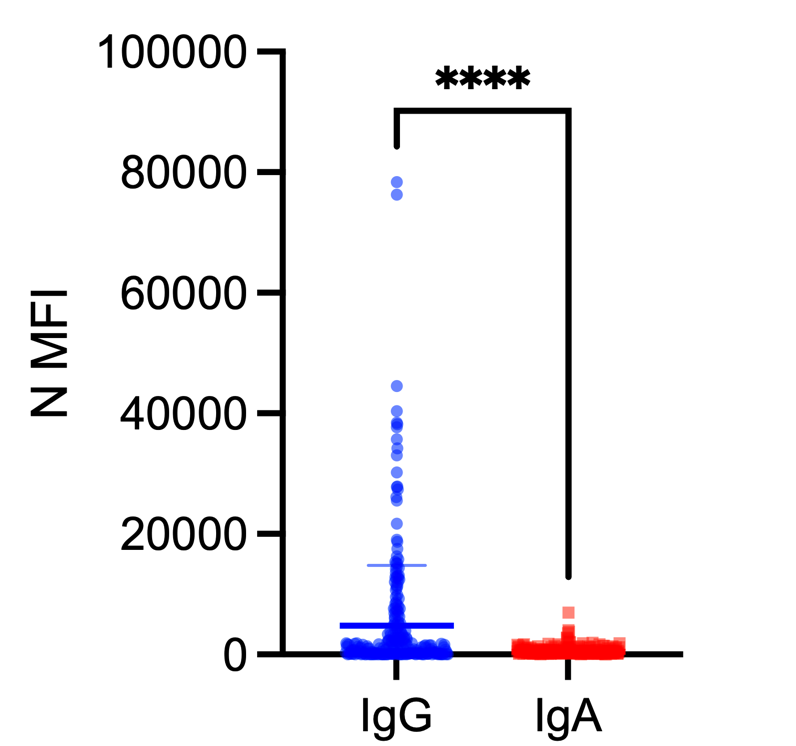

Supplement: S6 Fig — Comparison of nucleocapsid (N) protein-specific serum IgG and IgA outcomes (MFIs) among the study participants (mean and standard deviations). **** = p<0.0001, Welch’s t-test. N, Nucleocapsid Protein. (TIFF) [file pone.0307568.s006.tiff]

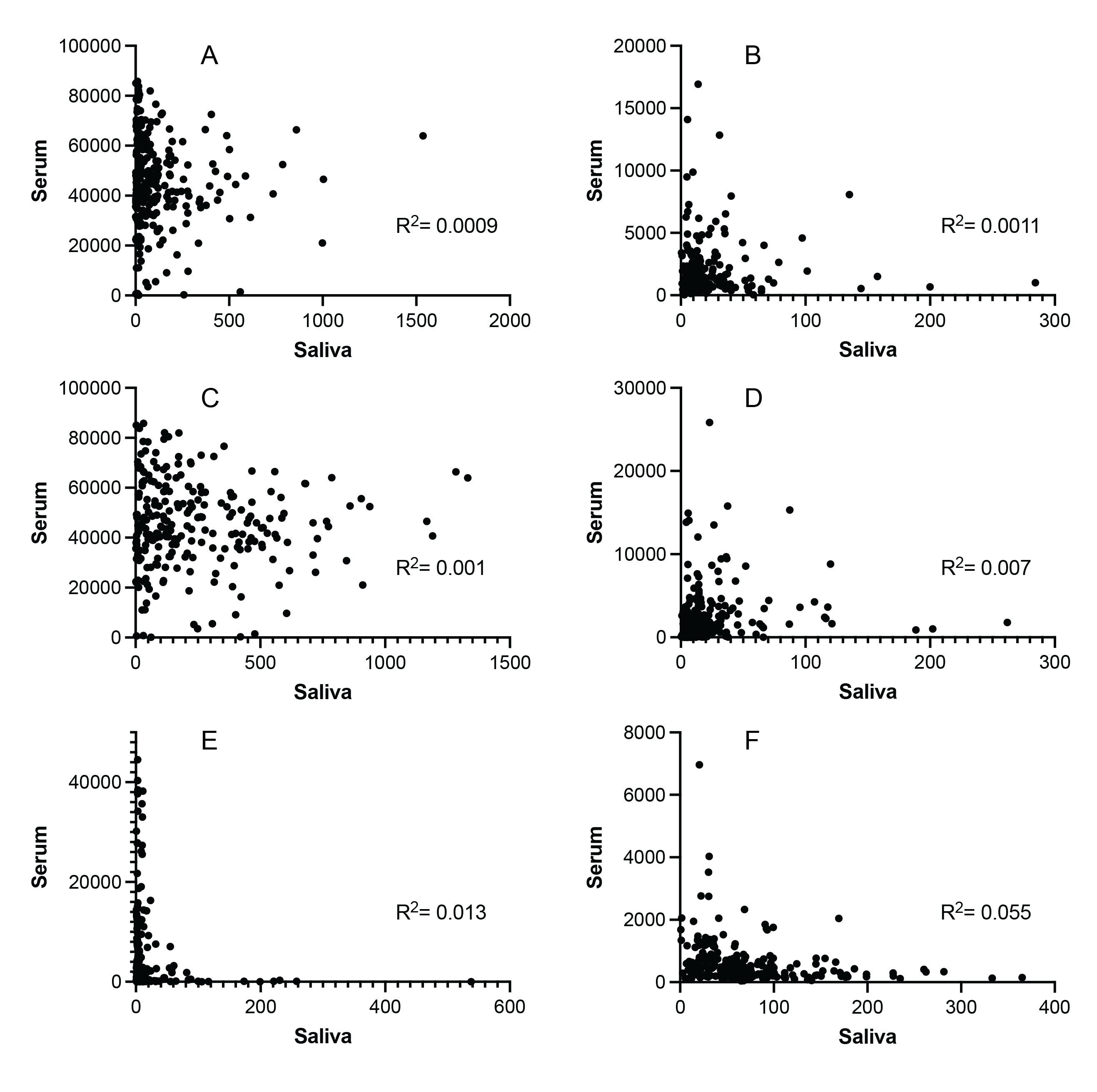

Supplement: S7 Fig — Line up of saliva versus serum comparisons of antigen-specific outcomes (MFI minus BSA for serum and transformed MFI for saliva [antigen- and isotype-specific MFI minus BSA, divided by total Ig, multiplied by 1000]), for anti-SARS-CoV-2 receptor binding domain (RBD; A, B), spike (S; C, D), and nucleocapsid (N; E, F) IgG (left column) and IgA (right column) antibody measurements. The outcomes between serum and saliva did not correlate for any antigen or isotype combination. (TIF) [file pone.0307568.s007.tif]

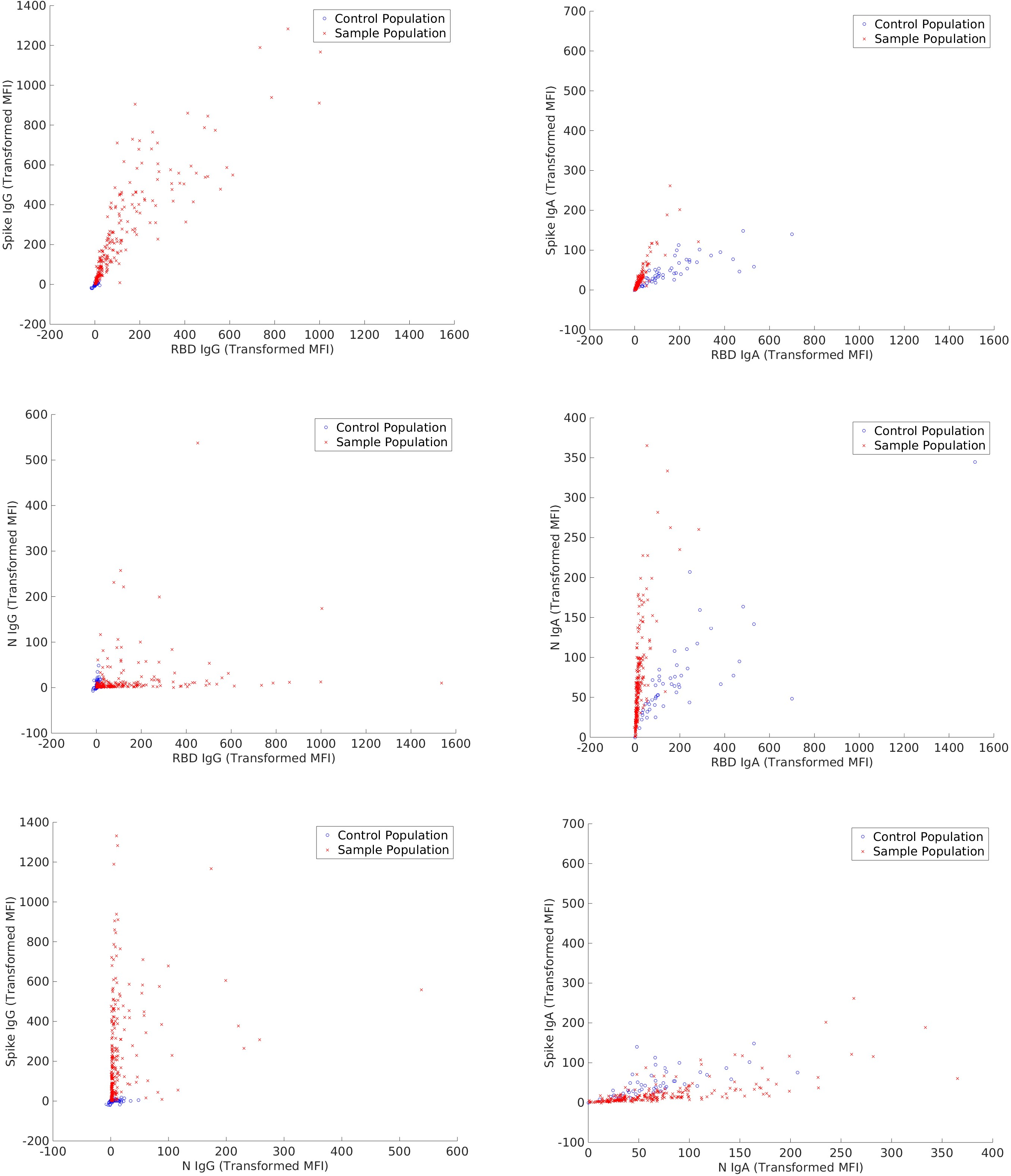

Supplement: S8 Fig — Comparison of study and control population by line up of saliva-based antigen-specific transformed MFI (antigen- and isotype-specific MFI minus BSA, divided by total Ig, and multiplied by 1000) for IgG (left column) and IgA (right column). For saliva IgG, the control sample population (sample collection method-matched samples from Kenya) always clusters in low MFI area and separate well from the study sample population (A, C, E), whereas for IgA the outcomes/MFIs from the control sample population overlap significantly with the study sample population for at least one antigen (B, D, F) and score higher maximum MFIs for RBD-specific outcomes (B, D). Hence, no saliva IgA percent seroprevalences could be calculated for the study samples based on these controls. S, Spike. RBD, Receptor Binding Protein. N, Nucleocapsid. (TIF) [file pone.0307568.s008.tif]

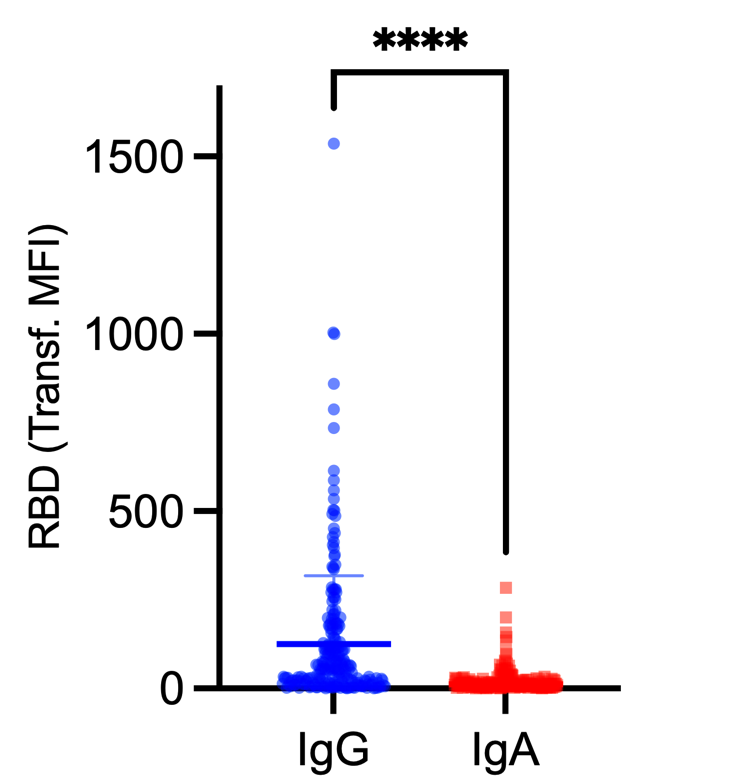

Supplement: S9 Fig — Comparison of RBD-specific saliva IgG and IgA outcomes (transformed MFI = [raw MFI/total Ig]*1000) among the study participants (mean and standard deviations). **** = p<0.0001, Welch’s t-test. RBD, Receptor Binding Protein. (TIFF) [file pone.0307568.s009.tiff]

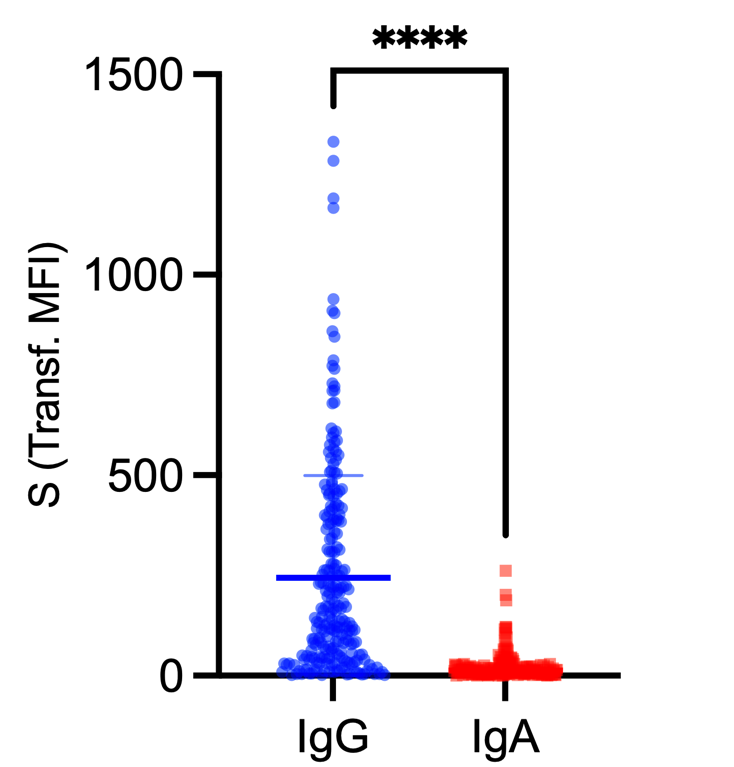

Supplement: S10 Fig — Comparison of S-specific saliva IgG and IgA outcomes (transformed MFI = [raw MFI/total Ig]*1000) among the study participants (mean and standard deviations). **** = p<0.0001, Welch’s t-test. S, Spike. (TIFF) [file pone.0307568.s010.tiff]

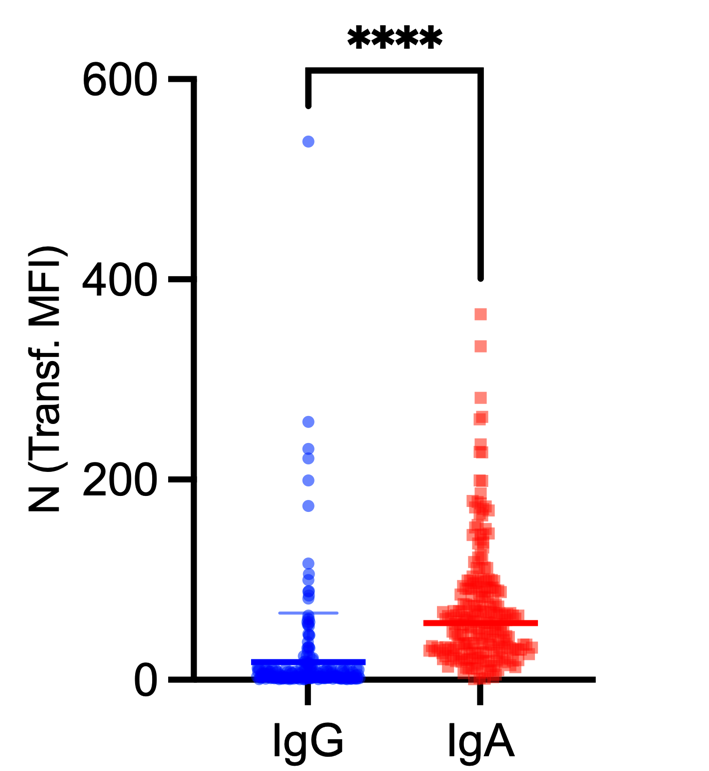

Supplement: S11 Fig — Comparison of nucleocapsid (N)-specific saliva IgG and IgA outcomes (transformed MFI = [raw MFI/total Ig]*1000) among the study participants (mean and standard deviations). **** = p<0.0001, Welch’s t-test. For N-specific outcomes in saliva, the IgA reads are higher compared to IgG. Whereas for RBD and S, the IgG reads in saliva are higher. Overall, the N-specific saliva IgG and IgA outcomes (transformed MFI) are lower than for RBD and S (i.e., lower overall MFI, see y-axis comparison between S8, S9, and S10 Figs). N, Nucleocapsid. (TIFF) [file pone.0307568.s011.tiff]
