# Supplementary material for: Minding the margins: Evaluating the impact of COVID-19 among Latinx and Black communities with optimal qualitative serological assessment tools
Source: PLoS One. 2024 Jul 25;19(7):e0307568. doi: 10.1371/journal.pone.0307568 (PMC11271856; doi:10.1371/journal.pone.0307568)
Supplement: S1 File — (PDF) [file pone.0307568.s012.pdf]

Active Seronet Recruitment

Record ID __________________________________

Participant ID __________________________________

Which language would you prefer to use?

¿Qué idioma prefiere usar?

- ◯ English
- ◯ Español

Demographic Information

Today's date __________________________________

First name __________________________________

Last name __________________________________

Your date of birth __________________________________
((mm/dd/yyyy))

Do you consider yourself.....?

- ☐ Black
- ☐ African American
- ☐ Chinese American
- ☐ Indian American
- ☐ Filipino American
- ☐ Asian
- ☐ Native Hawaiian or Other Pacific Islander
- ☐ American Indian or Alaska Native
- ☐ White
- ☐ More than one - Mix
- ☐ Other

Please explain other __________________________________

Do you consider yourself.....? __________________________________

What is your gender?

- ◯ Male
- ◯ Female
- ◯ Non-binary
- ◯ Other - please specify

Other - please specify __________________________________

Are you Latino or Hispanic?

- ◯ Yes
- ◯ No

What is the highest grade or level of school you have completed or the highest degree you have received?	◯ Primary/elementary school<br>◯ High school (or equivalent)<br>◯ College<br>◯ Advanced degree<br>◯ Other - please specify
Other - please specify	______________________
Your phone number	______________________<br>(Your contact information is collected solely for this research study and will not be shared with anyone outside of our team.)
Your email	______________________<br>(Your contact information is collected solely for this research study and will not be shared with anyone outside of our team.)
Age	______________________
Symptoms and Exposures	
Have you ever tested positive for the virus that causes COVID-19 (SARS-CoV-2)?	◯ Yes<br>◯ No
Other interval of testing	______________________
How often are you tested for the virus that causes COVID-19?	◯ Never<br>◯ Weekly<br>◯ Monthly<br>◯ When needed<br>◯ Other - please specify
Other - please specify	______________________
How many positive COVID-19/SARS-CoV-2 tests have you had?	______________________
When was the sample that turned out positive collected? (MM/DD/YYYY)	______________________<br>(If you don't know the exact date you tested positive, then please indicate the month and year.)

What symptoms did you experience in association with the positive test?	☐ Fever<br>☐ Fatigue<br>☐ Cough<br>☐ Loss of appetite<br>☐ Body aches<br>☐ Shortness of breath<br>☐ Mucus or phlegm<br>☐ Sore throat<br>☐ Headache<br>☐ Congestion or runny nose<br>☐ Nausea or vomiting<br>☐ Diarrhea<br>☐ Chills, sometimes with shaking<br>☐ Loss of smell or taste<br>☐ Purple fingers or toes (Chilblains)<br>☐ A rash on skin, or discolouration of fingers or toes<br>☐ Loss of speech or movement<br>☐ Conjunctivitis<br>☐ Unusual weakness<br>☐ Unexplained muscle aches<br>☐ Other Symptoms<br>☐ None
Other Symptoms	______________________
How many days were you sick for?	______________________
If you had to rate your overall well-being while sick where would it fall between 1 and 10? (10 being the most sick/worst symptoms)	○ 1<br>○ 2<br>○ 3<br>○ 4<br>○ 5<br>○ 6<br>○ 7<br>○ 8<br>○ 9<br>○ 10
Were you hospitalized because of these symptoms?	○ Yes<br>○ No
Have you had a positive test for SARS-COV-2 antibodies (serological/blood test for COVID-19)? Not counting the one being done today.	○ Yes<br>○ No
Was the antibody test done as part of a scientific study or was it by personal or clinical request?	○ Research Study<br>○ Personal (for example clinical reason)<br>○ Other
Other reason	______________________
Name of research study	______________________

When was the last time you had a positive antibody test? (mm/dd/yyyy)	__________________________ (If you don't know the exact date then please indicate the month and year.)
When was the antibody sample taken that came back positive? (mm/dd/yyyy)	__________________________ (If you don't know the exact date then please indicate the month and year.)
What symptoms did you experience in association with the positive antibody test?	☐ Fever<br>☐ Fatigue<br>☐ Cough<br>☐ Loss of appetite<br>☐ Body aches<br>☐ Shortness of breath<br>☐ Mucus or phlegm<br>☐ Sore throat<br>☐ Headache<br>☐ Congestion or runny nose<br>☐ Nausea or vomiting<br>☐ Diarrhea<br>☐ Chills, sometimes with shaking<br>☐ Loss of smell or taste<br>☐ Purple fingers or toes (Chilblains)<br>☐ A rash on skin, or discolouration of fingers or toes<br>☐ Loss of speech or movement<br>☐ Conjunctivitis<br>☐ Unusual weakness<br>☐ Unexplained muscle aches<br>☐ Other Symptoms<br>☐ None
Other Symptoms	__________________________
How many days were you sick for?	__________________________
If you had to rate your overall well-being while sick where would it fall between 1 and 10? (10 being the most sick/worst symptoms)	○ 1<br>○ 2<br>○ 3<br>○ 4<br>○ 5<br>○ 6<br>○ 7<br>○ 8<br>○ 9<br>○ 10
Were you hospitalized because of these symptoms?	○ Yes<br>○ No
Do you think you may have had COVID-19 even if not confirmed by a test?	○ Yes<br>○ No<br>○ I'm not sure
How many times do you think you may have COVID-19?	__________________________

When do you think you had COVID-19 (MM/DD/YYY)?

____
(If you don't know the exact date, then please indicate the month and year.)

What symptoms did you experience?

- ☐ Fever
- ☐ Fatigue
- ☐ Cough
- ☐ Loss of appetite
- ☐ Body aches
- ☐ Shortness of breath
- ☐ Mucus or phlegm
- ☐ Sore throat
- ☐ Headache
- ☐ Congestion or runny nose
- ☐ Nausea or vomiting
- ☐ Diarrhea
- ☐ Chills, sometimes with shaking
- ☐ Loss of smell or taste
- ☐ Purple fingers or toes (Chilblains)
- ☐ A rash on skin, or discolouration of fingers or toes
- ☐ Loss of speech or movement
- ☐ Conjunctivitis
- ☐ Unusual weakness
- ☐ Unexplained muscle aches
- ☐ Other Symptoms
- ☐ None

Other symptoms

____

How many days were you sick for?

____

If you had to rate your overall well-being while sick where would it fall between 1 and 10? (10 being the most sick/worst symptoms)

- ◯ 1
- ◯ 2
- ◯ 3
- ◯ 4
- ◯ 5
- ◯ 6
- ◯ 7
- ◯ 8
- ◯ 9
- ◯ 10

Were you hospitalized because of these symptoms?

- ◯ Yes
- ◯ No

If you had COVID-19, are you feeling better or do you have lingering health concerns?

- ◯ I am feeling better
- ◯ I am not back to 100%
- ◯ I believe I have long COVID-19 symptoms (health problems 4 or more weeks after first being infected with the virus that causes COVID-19).
- ◯ I never had COVID-19

Before the COVID-19 pandemic were you a smoker or a vaper?

- ◯ Yes - smoker
- ◯ Yes - vaper
- ◯ No

Prior to the COVID-19 pandemic, did you have any of the following medical conditions?	☐ Alcoholism<br>☐ Diabetes type I<br>☐ Diabetes type II<br>☐ Obesity<br>☐ Chronic kidney disease<br>☐ Dementia<br>☐ Alzheimer's disease<br>☐ Chronic liver disease<br>☐ Other Chronic respiratory disease (e.g., COPD, Emphysema) (specify)<br>☐ Asthma<br>☐ Chronic oxygen requirement<br>☐ Coronary artery disease<br>☐ Epilepsy<br>☐ Multiple sclerosis<br>☐ Other Chronic Neurological condition (specify)<br>☐ Hepatitis<br>☐ Hypertension<br>☐ Congestive heart failure<br>☐ Cancer<br>☐ Immune deficiency/HIV/AIDS<br>☐ Autoimmune/Immunocompromised condition<br>☐ Psychological/psychiatric condition<br>☐ Substance Use Disorders<br>☐ Other chronic diseases (specify)<br>☐ None
Other Chronic respiratory disease (e.g., COPD, Emphysema) (specify)	__________________________________
Other Chronic Neurological condition (specify)	__________________________________
Other chronic diseases (specify)	____
Vaccination	
Have you been vaccinated against influenza in the past 5 years?	○ Yes<br>○ No
Have you been vaccinated against the virus that causes COVID-19?	○ Yes<br>○ No
Have you been confirmed or suspected to have COVID-19 prior to vaccination?	○ Yes<br>○ No
When did you get your first vaccine dose? (mm/dd/yy)	__________________________________<br>(If you don't know the exact date, then please indicate the month and year.)
Which vaccine did you get?	○ Moderna<br>○ Pfizer<br>○ Johnson & Johnson<br>○ Other
Other vaccine	__________________________________

Did you experience vaccine-associated symptoms with your first shot?	☐ None<br>☐ Arm soreness<br>☐ Arm swelling<br>☐ Fever<br>☐ Chills<br>☐ Fatigue<br>☐ Nausea<br>☐ Swollen lymph nodes<br>☐ Muscle pain<br>☐ Headache<br>☐ Other body aches<br>☐ Other
Other symptoms	__________________________________
How long did the symptoms persist?	◯ 24 hrs<br>◯ 48 hrs<br>◯ Other
Other	__________________________________
If you had to rate your overall well-being while experiencing the vaccine-associated symptoms for the first dose, where would it fall between 1 and 10? (10 being the most sick/worst symptoms)	◯ 1<br>◯ 2<br>◯ 3<br>◯ 4<br>◯ 5<br>◯ 6<br>◯ 7<br>◯ 8<br>◯ 9<br>◯ 10
Were you hospitalized because of these symptoms?	◯ Yes<br>◯ No
Did you get your second vaccine dose?	◯ Yes<br>◯ No<br>◯ My vaccine only requires 1 shot
When did you get your second vaccine dose? (mm/dd/yy)	__________________________________<br>(If you don't know the exact date, then please indicate the month and year.)

Would you get an approved COVID-19 vaccine if offered to you?	◯ Yes<br>◯ No<br>◯ I would like more information on the vaccine before making that kind of decision
Why would you not want to get vaccinated?	__________________________________
Have you had a booster?	◯ Yes<br>◯ No
Which vaccine type were you boosted with?	◯ Moderna<br>◯ Pfizer<br>◯ Johnson & Johnson<br>◯ Other
Other booster type	____________________________

When did you get the booster? (mm/dd/yy)	____________________________<br>(If you don't know the exact date, then please indicate the month and year.)
Can someone from this study contact you in the future to ask you other COVID-19-related questions?	◯ Yes<br>◯ No
If you do not mind, please indicate why you prefer not to be contacted by a study team member.	__________________________________
Please feel free to leave any message for the study team.	__________________________________

Please make sure to submit the survey.
Thank you very much for your time and help!
