# Supplementary material for: Minding the margins: Evaluating the impact of COVID-19 among Latinx and Black communities with optimal qualitative serological assessment tools
Source: PLoS One. 2024 Jul 25;19(7):e0307568. doi: 10.1371/journal.pone.0307568 (PMC11271856; doi:10.1371/journal.pone.0307568)
Supplement: Attachment — Submitted filename: Response to Reviewers_PONE-D-24-10270.pdf [file pone.0307568.s015.pdf]

Thank you very much for the thoughtful review of the manuscript titled "*Minding the margins: Evaluating the impact of COVID-19 among Latinx and Black communities with optimal qualitative serological assessment tools*". We greatly appreciate the reviewers' comments which greatly improved the manuscript. Please see below our point-by-point responses in blue.

Reviewer #1:

Summary:
The authors have made a detailed qualitative serological assessment to investigate the impact of COVID-19 among the Latinx and Black communities. After going through the manuscript, I have found that the parameters for participant recruitment, sample collection, and sample processing had been performed meticulously (as the study involved culturally sensitive-marginalized communities) and were lucidly justified in the manuscript. The following of concerned protocols regarding the handling of human samples and due approval of the concerned authority/ethical committee is appreciated. The survey was performed to answer mainly three important investigations, i.e. (i) the impact of COVID-19 and vaccine uptake among marginalized communities, (ii) the utility of using saliva for serosurveys, (iii) a comparison of the utility of a bead-based multiplex assay vs. a point-of-care (POC) test for SARS-CoV-2 antibody measurements, and (iv) demonstration of the benefit of developing and using classification boundary methods for optimal interpretation of serological assays. The main research techniques used in the study were: Multiplex Luminex assay, POC test, across-plate normalization, and Qualitative assessment using statistical analysis.

The following few points need to be highlighted by the authors to aid the readers in perceiving the statements made thereof.

1. Methods (Qualitative serological assessment)
The authors have reflected on the use of alternative control samples from a Kenyan study for qualitative serological assessment which is elaborated in the supplementary information. However, I feel the need to cite the reference of that particular Kenyan study in the methods section itself for the ease of the readers.

Answer: The study that collected the samples for alternative purposes has not been published elsewhere. Instead of citing a reference, we added more details about these samples in the Supplemental Material. "Banked saliva samples, collected with the same SuperSal2 devices, served as alternative saliva control group for the seroprevalence calculation. We obtained 50 de-identified banked adult saliva samples that were collected in September of 2020 at Ahero, Kisumu County, Kenya under the KEMRI-SERU IRB Protocol # 3918. The first laboratory-confirmed COVID-19 case in Kisumu County, Kenya was identified on June 9, 2020, and the first wave peaked in July 2020.(1)"

Results

2. Demographics and Vaccine/Infection History: The authors have elaboratively discussed the population demographics and the history of COVID-19 vaccinations in the Latinx and Black communities. As the study is highly based on the self-declaration of health status by individuals of these communities with a very limited sample size, I feel the need to mention/compare the demographics with previous studies of COVID-19 on the Latinx and Black communities (as some more references are available).

Answer: We have added the following information and references to the results section, comparing our results to previously published studies in terms of pre-existing health conditions among US minorities, see lines 169-172 in manuscript with track changes. "Of note, the self-reported pre-existing health conditions (particularly the high prevalence of hypertension, diabetes, and asthma) reflect health-related risk factors reported among US minorities in other studies and increase the vulnerability to COVID-19 associated complications. (11-13)"

3. Blood-based SARS-CoV-2 Antibodies test:
The POC test covered both SARS-CoV-2 N and S antigens, while the multiplex assay allowed measuring the presence of antibodies based on individual antigens and therefore distinguishing between vaccine and infection-induced antibodies. The POC test was found reliable for detecting the RBD/S antibodies measured by the multiplex assay. This section needs no further changes.

Answer: No action item indicated. Comment well received. Thank you.

4. Discussion:
As mentioned by the authors, the antigen-specific outcomes between serum and saliva did not correlate, even though the antigen-specific IgG seroprevalences aligned. However, the authors have been able to ascertain the use of saliva as a less-invasive and accessible sample. Furthermore, this research highlights the need of hCoV cross-reactivity to be evaluated for reliable SARS-CoV-2 serosurvey results.

Answer: No action item indicated. Comment well received. Thank you.

Final comment: This research investigation is of potential importance as it makes important remarks on the impact of the COVID-19 disease among marginalized ethnic races using serological assessment tools and also devices some qualitative assessment parameters that will serve as effective investigation methods for further large-scale population-based surveys. The authors were able to answer the questions addressed initially in the investigation. No significant grammatical/English corrections were noticed in the manuscript. The manuscript is suitable for publication with very minor changes as mentioned above.

Answer: No action item indicated. Comment well received. Thank you.

Reviewer #2:

Summary:
The authors show the feasibility of using saliva and matching blood samples to determine SARS-CoV-2 antibody seroprevalence among a predominantly female and Hispanic population. They found good correlation between self-reported vaccination status (most were vaccinated against COVID) and a POC SARS-CoV-2 antibody device they employed on-site during the study visit and a multiplex assay that was used to test both blood and saliva for SARS-CoV-2-specific and endemic coronavirus IgG and IgA with the exception for salivary IgA.

1. My main comment is that the authors should address the discrepancy they see between prior self-reported COVID-19 infection and discrepant classification by blood and maybe also saliva test. ~40% of participants reported having had COVID-19 (test confirmed, n = 121 participants) but about 50 of them did not test positive for anti-N IgG. Similarly, ~60 out of ~160 participants who reported not having had COVID-19 do test positive for anti-N IgG. It is a bit odd to stratify Table 1 by this outcome and then not address it.

Answer: Thank you for pointing out the lack of discussion around this discordance. We added additional text to the discussion, see lines 383-402 in manuscript with track changes.

2. Another comment is that while normalization of saliva antibody signals with total Ig CONCENTRATION is often used, normalization with MFI values only works if both, the pathogen-specific signal and the total Ig MFI signal are within the linear range of the assay. The non-transformed (raw) data is not provided.

Answer: We believe the reviewer is concerned about how we accounted for differential salivation flow rates among study participants for the antibody measurements (vs. the across plate normalization) and additional discussion/information has been added to the supplemental material, see below. Further, we now **added the raw MFI for each antigen/isotype combination and the plate-specific normalization factors to the final complete data set**. However, the pathogen-specific signal varied across individual participants and that range could not be artificially adjusted.

"Given that individual salivary flow rates may change based on circadian rhythm, stress, and sample collection method, controlling for across sample variation for saliva-based antibody measurements is essential. We divided the individual antigen/isotype specific saliva measurements by total Ig antibodies to account for that variation, as has been done by others before (and there are few other viable alternative methods) and assured that the total Ig MFI signal is not over- or under saturating the assay. We also compared multiplex-based anti-SARS-CoV-2 IgG and IgA antibody measurements in matched serum and saliva samples and were able to confirm that the IgG-based serological outcomes in serum and saliva aligned."

Some minor comments:

3. Line 235 "[…] indicating past infection rather than vaccination and mirrored self-reported exposures."
The percentage of self-reported infection may be similar to the percentage of anti-N positive blood samples but Table 1 appears to suggest that only ~50% of participants who reported a prior SARS-CoV-2 positive test also tested anti-N positive. Conversely, ~ one third of those who did not report a prior SARS-CoV-2 positive test had a anti-N positive result. The authors may want to acknowledge this here or omit the "mirroring" statement if addressed in the discussion.

Answer: Thank you for pointing this out. We modified the sentence and added additional text/information, see lines 383-402 in manuscript with track changes.

4. Line 242 As for SARS-CoV-2 variants, the delta variant had the most abundant antibodies among our study population, see Fig 1.
Higher MFI does not necessarily mean most abundant antibody. MFI signals are influenced by a lot of factors including quality of the antigen, orientation of immunogenic (antibody-binding sites) regions of the antigen on the bead, antigen density on the bead, orientation of the bound antibody on the bead, etc. The study took place during the Omicron wave and, as the authors mentioned, most participants were vaccinated (i.e., "primed" with the Wuhan strain). Consider acknowledging that MFI signal strength alone does not mean that most participants were infected with Delta or revise sentence accordingly.

Answer: Good point. We added additional text acknowledging that MFI signals are influenced by several factors, see lines see lines 254-258 in manuscript with track changes. "As for SARS-CoV-2 variants, the delta variant had the highest mean MFI and reached the highest maximum MFI read, see Fig 1. While antigen-specific MFI are influenced by antigen quality and steric hindrance this may reflect that SARS-CoV-2 delta variant antibodies were the most abundant among our study population."

5. Line 263 "[…] indicating that the POC test was reliably detecting the RBD/S antibodies measured by the multiplex assay."
Are the authors trying to say that the POC assay does only classify blood samples with high/higher anti-S MFI as positive? Please clarify. Also, a brief description about what is meant with 2/3 antigen positivity (S/N; RBD/N, etc.) would be helpful. I.e., positive for IgG to all antigens or at least one of them, etc.? It might also be helpful to indicate the multiplex assay outcome (for one of the algorithms?) in Figure 2, e.g., coloring the dots according to the multiplex assay result or by using different (larger) symbols according to result and/or adding a threshold for S MFI / RBD MFI / N MFI.

Answer: Thank you for pointing out the confusion. We made edits to make it clearer. We were looking at the concordance between the POC test results and qualitative multiplex assay outcomes in different antigen combinations, see lines 272-284 in manuscript with track changes. We believe that part of the problem was that it was not

clear that the POC test detects both SARS-CoV-2 N and S antigens, this has been clarified as well, see next comment.

6. Line 266 “negative for the POC test across all antigen combinations”
Does the POC test detect antibodies against multiple antigens?

Answer: Yes, the POC test detects both SARS-CoV-2 N and S antigens (see lines 237/8 in manuscript with track changes) and this is now also mentioned in the methods (lines 103/4 in manuscript with track changes) and results (see lines 283/4 in manuscript with track changes).

7. Line 300 While anti-SARS-CoV-2 IgA has been shown to resolve faster than IgG
Maybe “wane” or “decline” would be the better word?

Answer: We replaced the word “resolve” with “wane”. Thank you. “While anti-SARS-CoV-2 IgA has been shown to wane faster than IgG, mucosal and blood-based IgA may provide protection from infections.”

8. Line 350 “the first COVID-19 case in MA was confirmed on Feb 1st 2022”
Should this be 2020?

Answer: Yes, thank you for catching that error. It has been fixed.

9. Line 363 groups as a risk factor, although and comorbidities did not correlate with lack of COVID-9 symptom resolution,
Grammar? Delete “and” I assume.

Answer: Yes, thank you for catching that typo. It has been fixed.
