# Supplementary material for: Minding the margins: Evaluating the impact of COVID-19 among Latinx and Black communities with optimal qualitative serological assessment tools
Source: PLoS One. 2024 Jul 25;19(7):e0307568. doi: 10.1371/journal.pone.0307568 (PMC11271856; doi:10.1371/journal.pone.0307568)
Supplement: Attachment — Submitted filename: Response to Reviewers_PONE-D-24-10270_7 5 24.pdf [file pone.0307568.s016.pdf]

PONE-D-24-10270
July 5, 2024

Thank you for the detailed review and additional feedback for the manuscript titled "*Minding the margins: Evaluating the impact of COVID-19 among Latinx and Black communities with optimal qualitative serological assessment tools*". Please see below our point-by-point responses in blue.

1. Re-number supplemental tables and figures. In the current version, Table S5 is the first supplemental table referenced in the Supplemental methods while Figure S11 is the first supplemental figure referenced in the text on page 12. We streamlined the numbering for the supplemental figures. S11 was the second supplemental figure to be listed and it is now labeled S2, while all the other supplemental figure numberings have been adjusted. The reason that Table S5 is the first supplemental table referenced in the supplemental methods is because Table S1-S4 are listed first in the main manuscript.

2. Please check the names of companies and products. It should be Sino Biological rather than Sino Biology in Table S5. Thank you for catching that. The company name has been corrected and the other ones verified.

3. Please make sure to use consistent and logical terminology and definitions throughout the text. For example, please correct statements in Lines 98-101 and in 2nd paragraph in the Supplemental Methods. The POC test detects IgG and IgM antibodies to S and N antigens (anti-SARS-CoV-2 antibodies), rather than anti-immunoglobulin antibodies in blood samples. Also, this test does not detect S and N antigens in blood. The POC test description in the main text and supplemental method has been corrected and adapted.

4. Lines 358 – 361. It appears that previously infected ("exposed") individuals include those who tested seropositive to the N antigen as well as those seronegative individuals who had been diagnosed with SARS-COV-2 infection (Table 1). Yes, not all those who self-reported a positive test were also positive for N antibodies. See lines 372-88. "The percent of self-reported infections aligned with the N-based IgG seroprevalence outcomes in serum (N: 49.9 %) and saliva (N: 48.0 %). Hence, within two years and four months (the first COVID-19 case in MA was confirmed on Feb 1st, 2020 (24) and the study recruitment ended in July of 2022) about half our diverse study population had been exposed to SARS-CoV-2. While the percent of self-reported infections aligned with the N-based IgG serum and saliva seroprevalence results, we found that reporting a positive SARS-CoV-2 test was not necessarily linked to the presence of anti-N IgG antibodies (Table 1). This is likely due to the relatively short half-life of anti-N IgG antibodies. Others have found that anti-N antibodies start declining within one month post-positive PCR test with over half the study population testing seronegative within 6-7 months.(25, 26) Since our study was implemented 2+ years after the first local COVID-19 case and since antibody levels may range across individuals, it is likely that the antibody levels had dropped below the detection limits by the time we collected and screened the blood samples of these individuals. Similarly, we found that participants who did not report a confirmatory test were positive for anti-N IgG

antibodies. Given that 4% to 41% of SARS-CoV-2 infections may be asymptomatic (27), it is likely that these individuals may have been infected but were not aware or did not seek testing."

5. Lines 395 and 450. No data to compare sensitivity values of Luminex and POC tests is provided in the paper. Please provide such data or remove statements about Luminex assay being more sensitive. The sensitivity and specificity for the blood-based Luminex analyses was computed and listed in S2 Table and S3 Table in supplemental material but not for the POC. The sentences have been amended accordingly. See lines 303-306, 529-532 and 473/4.

6. Supplemental Information, Multiplex Assay – Saliva: Please confirm that undiluted saliva samples were tested for total IgG and IgA. The concentration of total Ig in saliva is so high that it would be reasonable to use highly diluted saliva in this analysis. Yes, as indicated in the first paragraph of the "Multiplex Luminex Assay – Saliva" section in the supplemental material, we used undiluted saliva to screen for anti-SARS-CoV-2 antibodies. While the total Ig in saliva was high (as expected/previously described), the MFI for the CoV-specific antibodies had a wide range (some low depending on the antigen measured), which led us to use undiluted saliva for the screening.
